# The γ-secretase complex: from discovery to a therapeutic target

**DOI:** 10.1039/d6cb00022c

**Published:** 2026-06-05

**Authors:** Shweta R. Malvankar, Michael S. Wolfe

**Affiliations:** a Department of Medicinal Chemistry, School of Pharmacy, The University of Kansas 1567 Irving Hill Road, GLH 2215 Lawrence KS 66045 USA mswolfe@ku.edu

## Abstract

γ-Secretase is an intricate intramembrane aspartyl protease that cleaves within the transmembrane domain of ∼150 substrates and is considered the ‘proteasome of the membrane’. This enzyme consists of four different subunits, with presenilin being the catalytic subunit. This review provides a brief overview of γ-secretase as a proteolytic enzyme, from its biochemistry and biology to its roles in disease and potential as a therapeutic target. A detailed discussion on the discovery and structure of γ-secretase is followed by a survey of its substrates, including the most studied amyloid precursor protein and the Notch1 receptor, and a description of substrate processing and sequence specificity. The role of γ-secretase in human biology and pathology is also detailed, with a particular focus on Alzheimer's disease (AD), in which the pathogenicity of the γ-secretase product amyloid-β peptide is still a matter of controversy. Lastly, the potential of γ-secretase inhibitors and modulators for the treatment of AD and other diseases is considered.

## Abstract 2

γ-Secretase is an intricate intramembrane aspartyl protease that cleaves within the transmembrane domain of ∼150 substrates, plays critical roles in biology and disease, and is considered the ‘proteasome of the membrane’.

## Graphical abstract

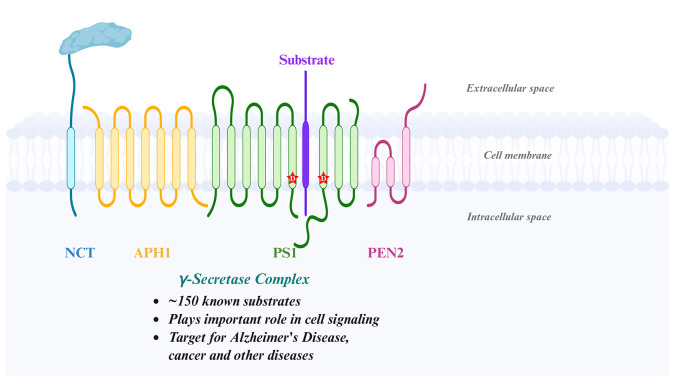

## Intramembrane proteases

Intramembrane proteases (IMPs), also known as intramembrane-cleaving proteases (I-CLiPs), are transmembrane enzymes, with their active site located within the hydrophobic environment of the lipid bilayer.^1^ IMPs show a range of substrate specificities but all cleave within the transmembrane domains of their substrates. Although mechanistically similar to water-soluble proteases, IMPs are not evolutionarily related to these classical proteases. Moreover, the catalytic rates of IMPs are extremely slow.^2–5^ IMPs cleave membrane protein substrates within their transmembrane regions by a process called regulated intramembrane proteolysis (RIP), which is conserved from bacteria to humans.^6,7^ RIP involves two regulated aspects, the first being ectodomain shedding of the substrates prior to intramembrane proteolysis by ‘sheddases’ (with the exception of rhomboid serine protease), and the second being transport/trafficking of the involved enzymes or their substrates.^8,9^ IMPs create an environment within the lipid bilayer that is suitable for water and hydrophilic residues to conduct hydrolysis of their substrates.^10^ These enzymes are essential in biology,^10^ and IMP-mediated cleavage events are often signaling mechanisms, such as in the Notch signaling pathway and EGF (epidermal growth factor) pathway. Impaired functioning of IMPs occurs in various pathological conditions, including Alzheimer's disease (AD), cancer, Parkinson's disease (PD), diabetes, and more.^11,12^ Many IMP members have been identified since their discovery in 1997.^13^ Based on the catalytic mechanism, the four classes of known IMPs are metalloproteases, such as site-2 protease (S2P); rhomboid serine proteases; glutamyl IMPs, such as Rce1; and aspartic IMPs, such as γ-secretase and signal peptide peptidase.^10,14–18^ This review discusses in detail a founding member of the IMP family, γ-secretase, along with its many substrates, roles in human diseases, and therapeutic potential.

## Discovery and components of the γ-secretase complex

Plaque deposits of the 4-kDa amyloid β-peptide (Aβ) are found in the brains of Alzheimer's disease (AD) patients. This fragment is generated from the amyloid precursor protein (APP) through successive cleavage by two proteases: β-secretase and γ-secretase.^19^ γ-Secretase is the most biochemically complicated of the IMPs.^20^ The proteolytic activity that produces Aβ from APP was first described as “γ-secretase” in relation to AD more than three decades ago;^21,22^ however, the enzyme and its components were not fully identified until a decade later.^23^ Around the same time, missense mutations associated with dominantly inherited familial AD (FAD) were found in APP^24,25^ as well as in presenilin-1 and -2 (PS1 and PS2).^26,27^ PS1 activity was soon linked to Aβ as FAD PS1 mutations were shown to alter its production.^28,29^ The discovery that PS1 deficiency led to a substantial reduction in Aβ production suggested that PS1 mediates most proteolytic cleavage of APP, with the remainder cleaved by PS2.^30–32^ Presenilin FAD mutations were found to elevate the proportion of the Aβ42 variant relative to Aβ40.^28,33–36^ All these observations regarding presenilin were made in the context of γ-secretase. Cell-based assays for Aβ production were used to test peptidomimetics as inhibitors of γ-secretase, and these inhibitors suggested that the enzyme is an aspartyl protease.^37^ It was then discovered^38^ and further confirmed^3,9,40^ that two conserved transmembrane aspartates in presenilins are critical for γ-secretase cleavage activity. Transition-state analog peptidomimetic inhibitors of γ-secretase were subsequently found to bind with the presenilin active site.^41,42^ Simultaneously, it was discovered that the presenilin-dependent γ-secretase activity that is responsible for APP cleavage is also involved in and crucial for transmembrane cleavage of the Notch1 receptor to release the Notch intracellular domain (NICD), a second messenger for cell signaling.^43–49^ All these discoveries collectively identified presenilin as a membrane-embedded aspartyl protease, the catalytic component of γ-secretase.

**Fig. 1:**
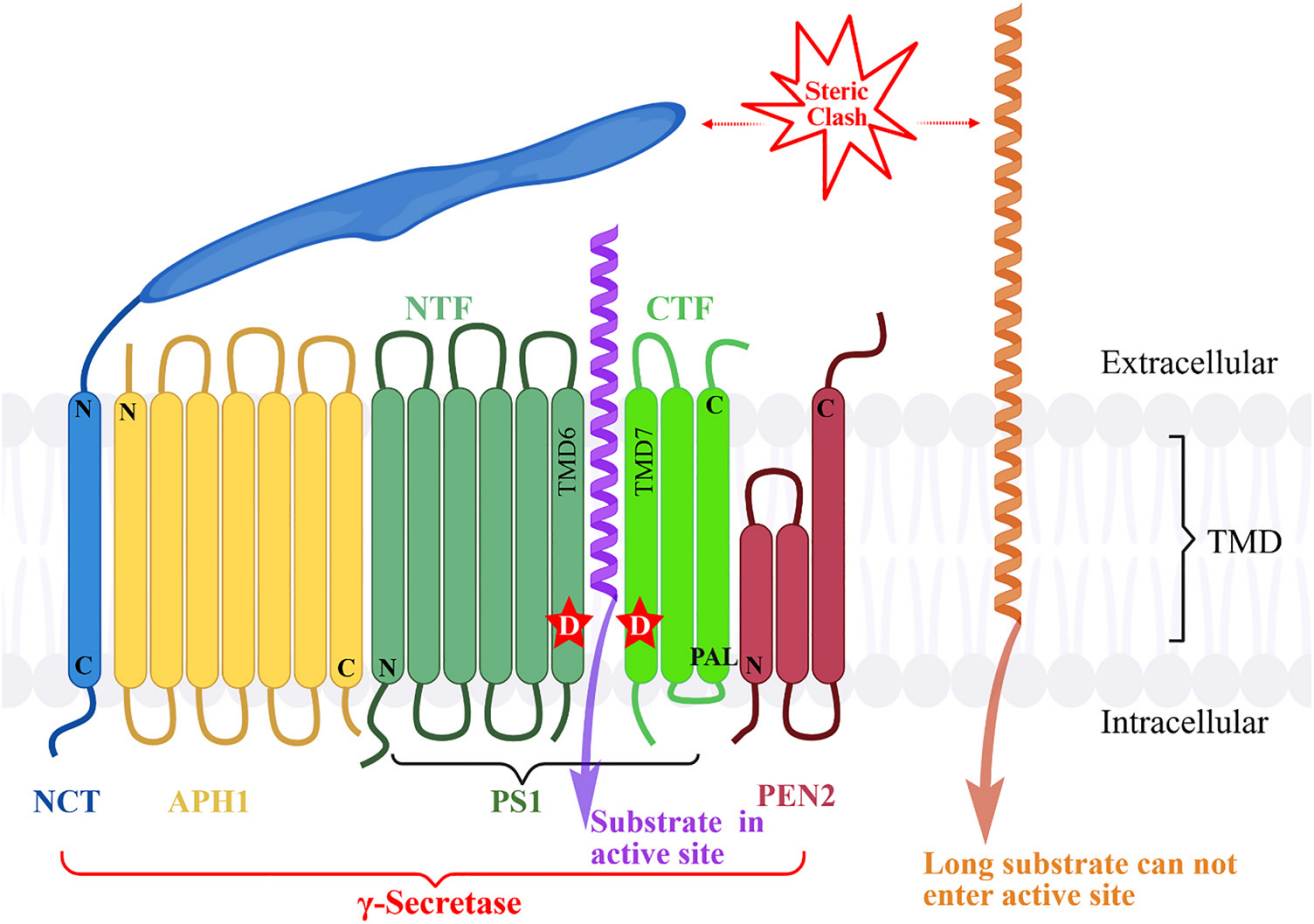
γ-Secretase complex and substrate interaction. γ-Secretase complex containing all its four components NCT (blue), APH-1 (yellow), Pen-2 (red) and catalytic PS1 NTF and CTF (dark and light green, respectively). Catalytic aspartates are marked by D in TM6 and TM7 of PS1. The substrate entry in the active site is guided by NCT in the extracellular region. The short substrate (purple) can enter the active site, while the longer substrate (orange) cannot because of the steric clash between the long ectodomain of the substrate and extracellular NCT. NCT: nicastrin, APH1: anterior pharynx defective-1, PEN2: presenilin enhancer 2, PS1: presenilin1, PAL: P_433_A_434_L_435_ motif, N: N-terminal, C: C-terminal, TMD: transmembrane domain, NTF: N-terminal fragment, and CTF: C-terminal fragment.

Presenilin is expressed in the endoplasmic reticulum (ER) and undergoes proteolysis to form N-terminal and C-terminal fragments (NTF and CTF, respectively).^50–52^ These fragments were found to remain associated within a higher molecular weight complex,^53–55^ suggesting that presenilin is a part of a larger complex. When the two conserved transmembrane aspartates were discovered to be essential for γ-secretase activity, they were also found to be required for presenilin cleavage into NTF and CTF, indicating presenilin is a zymogen that cleaves itself into its active form.^38^ Later, three additional γ-secretase components were identified through biochemical and genetic studies as nicastrin (NCT),^56,57^ anterior pharynx defective 1 (APH-1)^5,8–60^ and presenilin enhancer 2 (PEN-2).^61,62^ All four membrane protein components assemble together in 1 : 1 : 1 : 1 stoichiometry, with presenilin undergoing proteolysis to NTF and CTF to form the proteolytically active γ-secretase complex.^63–66^ No additional proteins were found to be stably connected with the complex. Enzyme activity is regulated mainly by its primary components; however, additional regulation may be provided by lipid composition and by other associated (non-essential) protein factors that may modulate the enzyme complex.^67,68^ The complex of the γ-secretase enzyme is depicted in Fig. 1.

Presenilin has nine transmembrane domains (TMDs),^69^ with the two catalytic aspartates (D257 and D385 in PS1) residing in TMD 6 and 7.^38^ The C-terminally conserved ‘P_433_A_434_L_435_’ motif in TMD9 of PS1 is an important part of the active site of PS1 and is also important for the proteolytic activity of the enzyme.^70–72^ The PAL motif is essential for PS1 endoproteolysis^70^ and contributes towards the proper active-site conformation of the enzyme.^71^ Another motif, GxGD, which contains one of the two catalytic aspartates (D385), is critical for substrate specificity, selectivity and proteolytic activity of γ-secretase.^73,74^

**Fig. 2:**
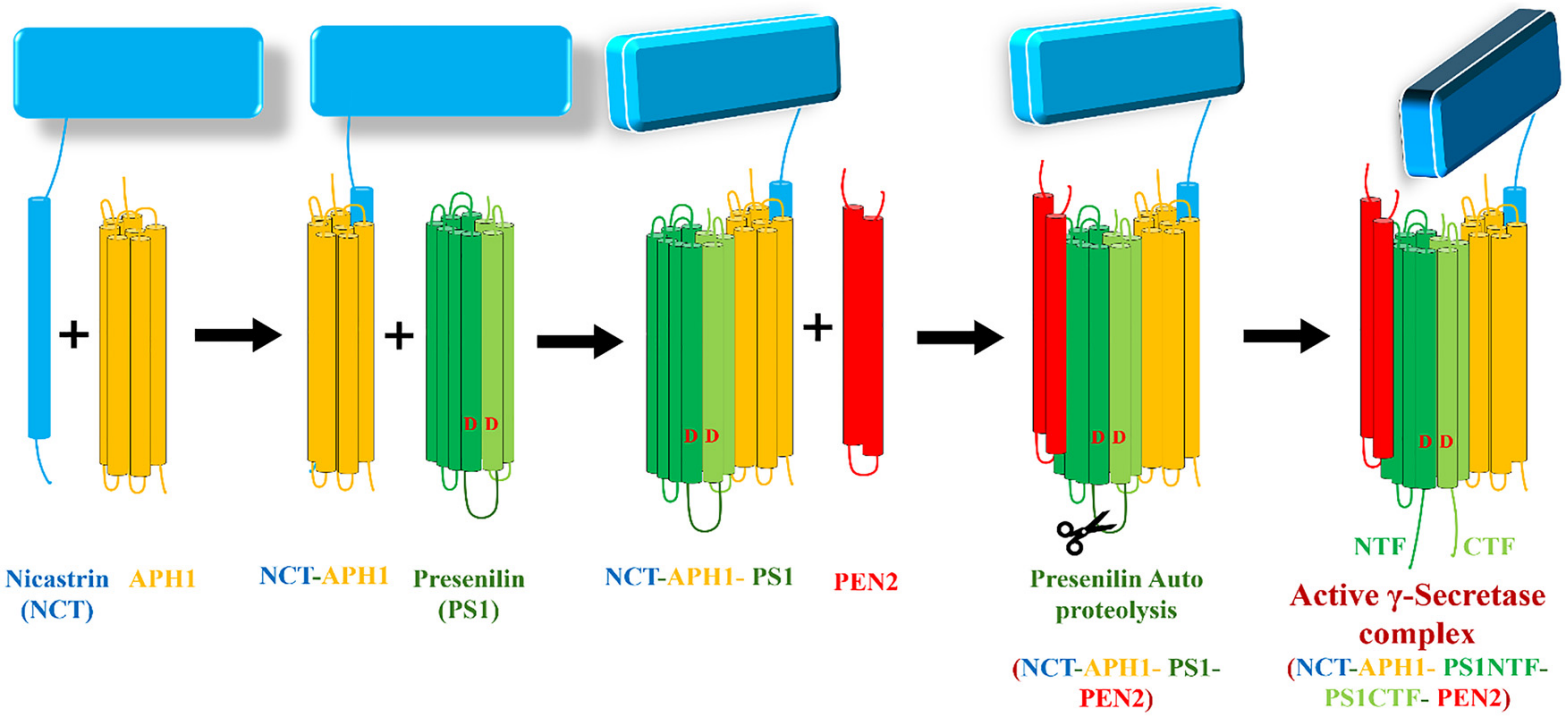
Sequential assembly of γ-secretase components into the full active complex All four γ-secretase components of the enzyme assemble in a sequential manner to form the full complex, which is activated by autoproteolysis. First nicastrin (NCT, blue) and APH-1 (yellow) come together to form the NCT–APH1 complex. Presenilin1 (PS1, dark and light green) associates itself with the NCT–APH1 complex, forming the NCT–APH1–PS1 complex. PEN-2 (red) then interacts with TMD4 of PS1 in the complex, forming the NCT–APH1–PS1–PEN2 complex. The interaction of PEN-2 and PS1 causes autoproteolysis of PS1 in NTF and CTF, generating the fully activated γ-secretase complex. Catalytic aspartates are marked by D in TM6 and TM7 of PS1.

The other components of γ-secretase are essential for forming the mature enzyme complex.^75^ NCT has only one TMD, a small intracellular and a large extracellular domain.^56^ It plays a role in substrate recognition^76^ and their selective recruitment through steric hindrance.^77^ In contrast, another report says that NCT helps stabilize the enzyme complex but is not essential for substrate recognition.^78^ NCT is essential for APP processing and is critical but not absolutely required for Notch processing.^79^ The role of APH-1 is apparently to stabilize the enzyme complex, serving as a scaffold for assembly, while PEN-2 is essential for endoproteolytic processing of PS1.^23,65,80^ APH-1 has seven TMDs, with the C-terminal end facing the cytosol and the N-terminal end facing the extracellular space,^81^ and is reported as not being absolutely required for processing of APP or Notch substrates.^79^ PEN-2 was predicted to have only two TMDs, with both the C- and N-terminal ends facing the extracellular region and a connecting loop in the cytosol.^82^ PEN-2 is also known to be involved in maturation and stabilizing the enzyme complex.^83,84^ All four components need to be assembled in the correct order for the enzyme to be fully active. In the endoplasmic reticulum, NCT and APH-1 first bind together, which allows binding of full-length PS to the complex, followed by PEN-2. TMD4 of PS1 interacts with PEN2 in complex formation,^85^ which then triggers autoproteolysis of PS1 into NTF and CTF. Furthermore, the TMD1 sequence of PEN2 (specifically the proximal 2nd/3rd part) is essential for autoproteolysis of PS1.^86^ The complex then travels to the Golgi for glycosylation,^75,87^ and further traffics through the secretory pathway to the cell surface and to endosomes and lysosomes. The sequential assembly of all the components to form the active enzyme γ-secretase complex is shown in Fig. 2.

## Structure of the γ-secretase complex

Cryo-electron microscopy (cryo-EM) images of γ-secretase have provided detailed insights into the structure of the protease complex and its interaction with substrates over the last decade. The first high-resolution cryo-EM structure of intact γ-secretase, determined at 4.5 Å resolution,^88^ revealed 19 TMDs arranged in a horseshoe shape. The active site is located on the convex side of this horseshoe-shaped TMD complex, with the NCT ectodomain positioned above the PS active site, serving as a gatekeeper to allow entry of only substrates with short ectodomains (Fig. 2).^77,88–90^ Structural organization of the enzyme subunits and their TMDs that were proposed based on biochemical studies were confirmed by these cryo-EM studies. PS1 is central in the complex, with its NTF attached to PEN2 and CTF interacting with APH1, which interacts with the lone TM of NCT.^91^ Cryo-EM studies further clarified that γ-secretase has 20 TMD instead of 19. Specifically, PEN2 contains three TMDs, in contrast to the previously proposed two.^82^ TM2 of PEN2 only goes into the membrane halfway through and turns back into the cytoplasm, localizing its N-terminus into the cytoplasm, whereas the C-terminus is localized towards the extracellular side.^91^ The poorly resolved TMD2 of PS1 suggested that TMD2 is flexible and involved in the lateral entry of substrate TMD to the active site. Subsequently, substrate binding is proposed to cause conformational changes in the enzyme that activate the two catalytic aspartates by bringing them into proximity.^87^ Additionally, part of TMD6 was also unresolved, presumably because of flexibility, suggesting that, together with TMD2, it likely regulates substrate entry.^92^

Subsequent cryo-EM structures of γ-secretase bound to substrates APP and Notch, at 2.6–2.7 Å resolution, provided further details of enzyme interactions with specific substrates.^93,94^ These studies revealed that substrates acquire a hybrid α-helical/β-sheet structure that exposes the cleavage site in the active site and support a substrate-helix unwinding model in the active site of γ-secretase for their cleavage. Furthermore, the lateral diffusion model for substrate entry and involvement of TMD2 and part of TMD6 of PS1 was also confirmed, as these two regions were well resolved upon substrate binding.

The γ-secretase structure bound to the dipeptide analog inhibitor DAPT (*N*-[*N*-(3,5-difluoro-phenacetyl)-l-alanyl]-*S*-phenylglycine *tert*-butyl ester) showed the inhibitor binding close to the active site. The structure also suggested that DAPT binding induces a conformational change in PS1 that does not allow entry of the substrate, thereby inhibiting enzyme activity.^95^ Recent structures of the protease bound to three other inhibitors (semagacestat, avagacestat, and the peptidomimetic transition state analog L685,458) showed that all three γ-secretase inhibitors (GSIs) interact with the same site on PS1 occupied by the APP or Notch β-strands in and around the active site, hence inhibiting substrate binding.^96^ In contrast, the structure of γ-secretase bound to E2012 revealed that this γ-secretase modulator (GSM) binds extracellularly to an allosteric site on the protease complex.^96^

Computational molecular dynamics (MD) studies have also provided useful insights into the mechanism and dynamics of substrate–enzyme interactions, substrate cleavage, conformational changes, and enzyme inhibition and modulation.^97,98^ For example, combining MD and biochemical studies helped understand APP substrate processing by γ-secretase^99^ and develop a model of γ-secretase-Notch complexes for Notch wild-type and mutant cleavage.^100^ In the latter study, an incorrect registry of Notch1 binding was identified in the cryo-EM structure, resolving discrepancies with biochemical results by systematic replacement of bound APP residues with corresponding Notch residues. The model developed through this “replacement method” was highly consistent with biochemical results.^100^

## Various substrates of γ-secretase

γ-Secretase is currently known to cleave more than 145 substrates, including APP and the Notch1 receptor.^101^ The products generated from these cleavage events have varying functions. Most of the substrates are type I integral membrane proteins^90^ with long ectodomains that require shedding before γ-secretase processing.^102^ γ-Secretase cleavage of substrates is not dependent on a particular consensus sequence; however, short extracellular domains are required for higher cleavage efficiency (Fig. 1).^77,103^ Because of the different isoforms of PS and APH found in humans, multiple enzyme complex subtypes have been identified.^67^ Based on their locations and expression levels in the human body, different substrates may be cleaved by different complexes.^101^

**Fig. 3:**
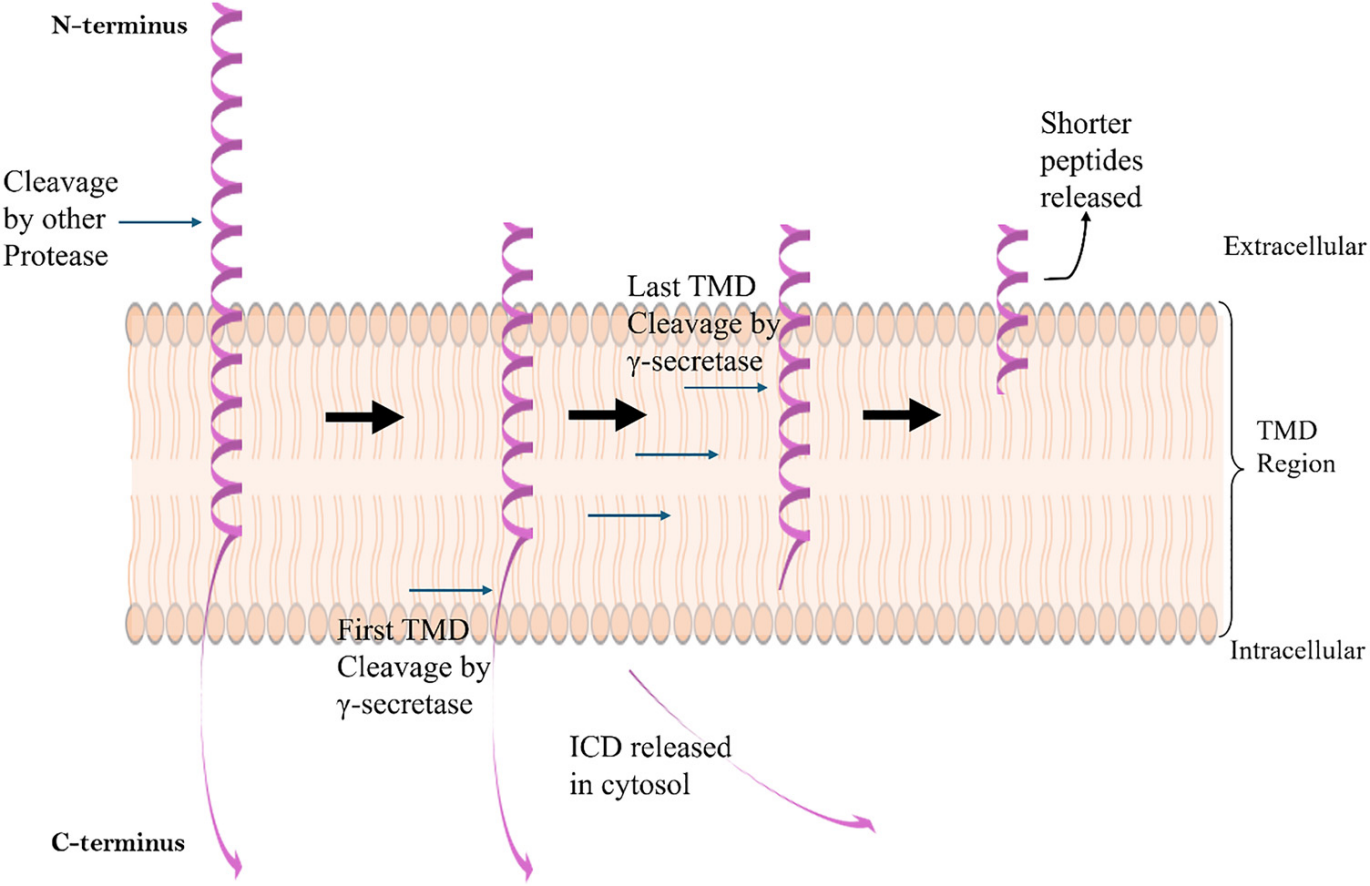
General process of substrate cleavage by γ-secretase. The type-1 transmembrane domain (TMD) substrate with a long ectodomain is first cleaved by a protease close to the surface of the TMD, generating a shorter membrane-bound fragment that enters the active site of the enzyme. γ-Secretase then cleaves at the ε-site first, near the cytosolic end, and releases the C-terminal cleavage product (intracellular domain: ICD) into the cytosol. Then, the remaining membrane-bound fragment is trimmed further and released into the extracellular space as an N-terminal product.

Although some common features of most γ-secretase substrates are noted,^68^ the enzyme promiscuously cleaves various substrates, and there is no known consensus sequence.^104^ Moreover, in cases of substrates such as Notch,^47^ APP,^105^ and CD44,^106^ proteolytic processing by γ-secretase generates multiple products with varying C-terminal ends. Hence, the enzyme has been dubbed ‘the proteasome of the membrane’, cleaving many substrates within their TMDs and playing critical roles in biology and medicine.^107^ The enzyme has a substrate-binding exosite at the PS1 NTF/CTF interface that is distinct from, but proximal to, the active site.^108^ The first (endoproteolytic or ε) cleavage occurs near the cytosolic interface of the membrane, releasing the fragment called the intracellular domain (ICD) into the cytosol (Fig. 3). Although a wide range of substrates are known for the enzyme, specific cleavage sites have been identified for very few. In this review, we will discuss two well-studied γ-secretase substrates, APP and Notch1, in detail and briefly touch upon other substrates, followed by a discussion of a substrate cleavage model and sequence specificity.

### Amyloid precursor protein (APP)

**Fig. 4:**
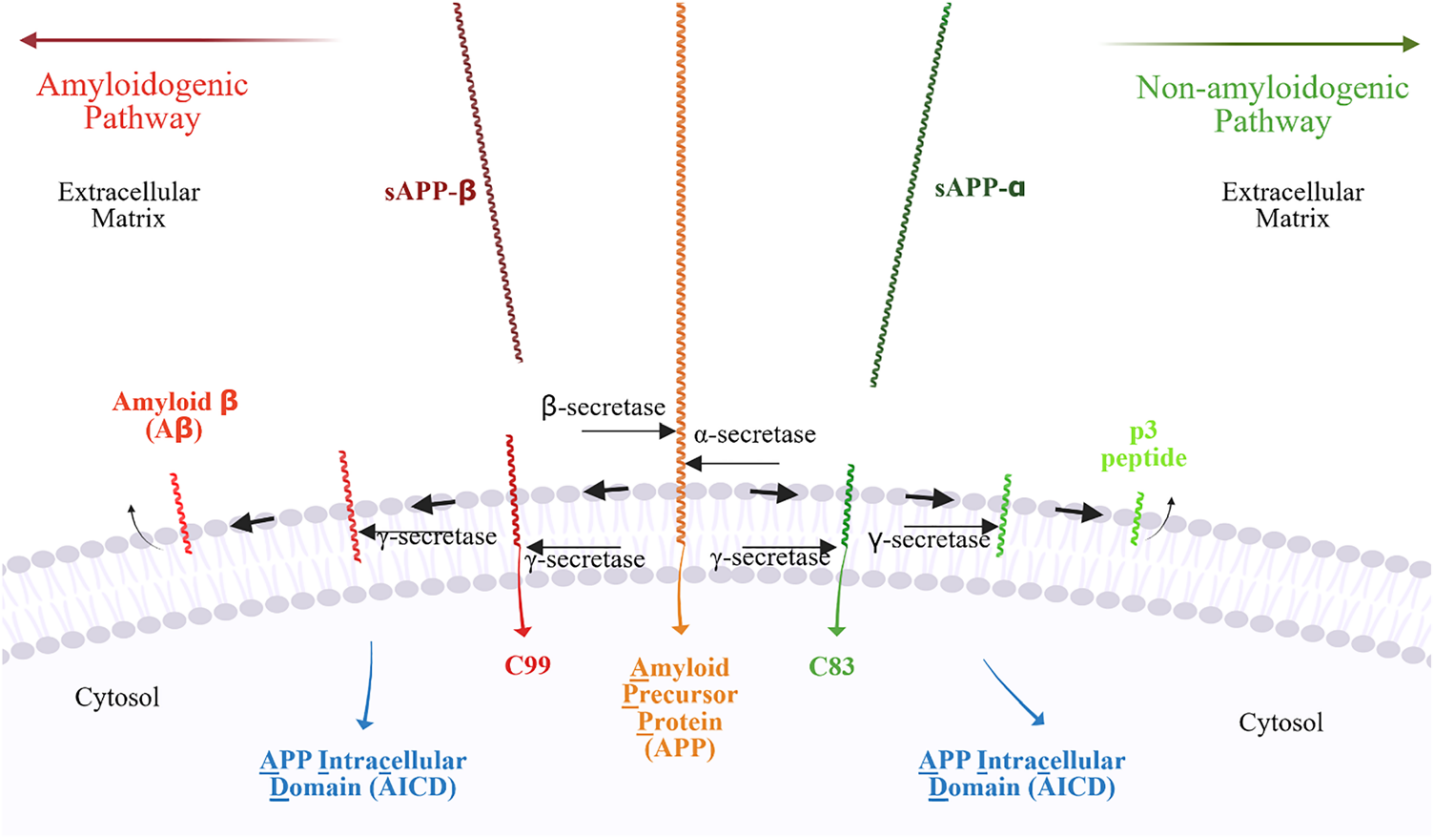
Proteolysis of full-length APP by amyloidogenic (red) and non-amyloidogenic (green) pathways. The long ectodomain of APP is cleaved by either α- or β-secretase through extracellular shedding. The membrane-bound C99 is then cleaved by γ-secretase to release the AICD fragment into the cytosol. Membrane-bound longer Aβ fragments are then further trimmed down by γ-secretase to release shorter fragments into the extracellular space.

Amyloid precursor protein (APP) is a single-pass conserved type I integral membrane protein. The APP gene is located on chromosome 21, and tissue-dependent alternate splicing leads to expression of three major isoforms, of which APP695 is almost exclusively found in the brain.^109,110^ Two other homologous APP-like proteins (APLP-1 and APLP-2) are known; however, they differ in sequence in the Aβ region.^111^ The proteolysis of full-length APP involves multiple cleavages by proteases and can occur by two different pathways: amyloidogenic and non-amyloidogenic, as shown in Fig. 4.

The amyloidogenic pathway (Fig. 4) starts with the juxtamembrane cleavage of full-length APP by a pepsin-like aspartyl protease, β-secretase (also known as BACE1: β-site APP-cleaving enzyme), releasing the soluble extracellular domain called sAPP-β and leaving behind a membrane-anchored stub containing 99 amino acids called the β-carboxy-terminal fragment (β-CTF or APP-C99).^112–117^ C99 then undergoes cleavage by γ-secretase within its single TMD, generating amyloid β-peptide (Aβ) and the APP intracellular domain (AICD). Recent reviews suggest that C99, rather than Aβ, may be a culprit that accumulates in AD and acts as an early pathogenic trigger.^118,119^ The γ-secretase processing of C99 is discussed in further detail later.

Alternatively, in the non-amyloidogenic pathway (Fig. 4), the full-length APP is cleaved within the extracellular region of the Aβ sequence by α-secretases, ADAM-like metalloproteases, to release a longer soluble ectodomain called sAPP-α and leaving behind a membrane-bound fragment of 83 amino acids called C83 (or α-CTF).^117,120^ The latter is further cleaved by γ-secretase to generate AICD and an N-terminally truncated Aβ-like peptide, dubbed p3.^117^ The α-secretase cleavage of APP is the most abundant pathway in cells.^120^ APP and its various proteolytically derived fragments have been proposed to have different biological roles, which are reviewed in detail elsewhere.^109–111^

### Notch receptor family

Notch receptors are another type I integral membrane protein that are arguably the most important substrates of γ-secretase. The *Notch* gene was first identified in *Drosophila*, encoding a 300 kDa protein. In mammals, four Notch isoforms Notch1–4 are present and structurally similar to *Drosophila* Notch and *C. elegans* lin-12 and glp-1.^121^ These evolutionarily conserved cell-surface receptors are crucial for the development and health of all metazoans, as they are involved in cell proliferation, homeostasis and damage repair.^122^

**Fig. 5:**
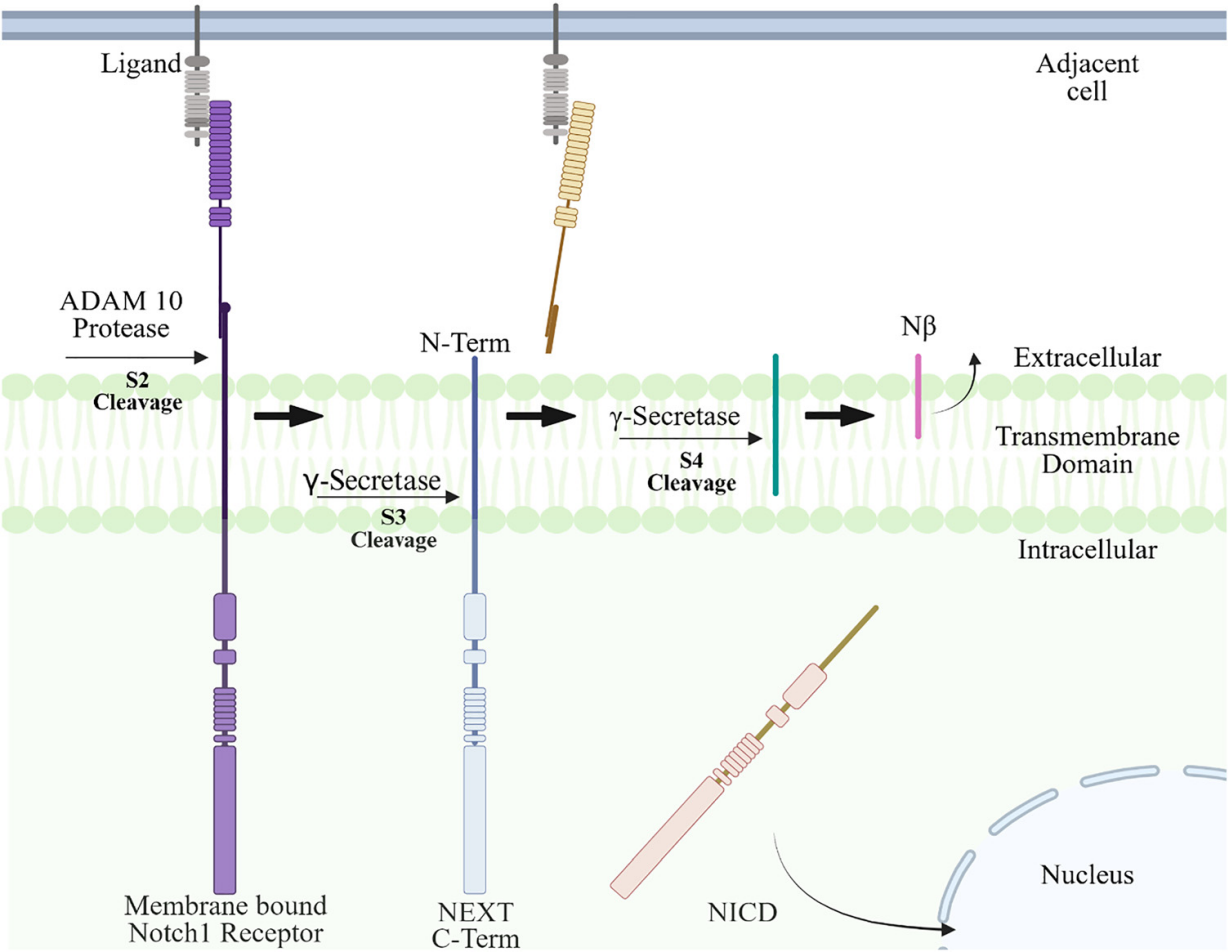
Process of Notch1 proteolysis by γ-secretase. Membrane-bound full-length Notch1 receptor (purple) is cleaved by ADAM 10 protease after binding with the ligand from an adjacent cell, generating NEXT (Notch extracellular truncation). NEXT is then cleaved by γ-secretase at the S3 cleavage site, releasing NICD (Notch intracellular domain) that traverses to the nucleus for gene expression. The remaining membrane-bound Notchβ (Nβ) is further trimmed by γ-secretase to release smaller Nβs into the extracellular space.

Aberrant Notch signaling can cause various cancers and other diseases.^123,124^ Notch protein has a large extracellular domain that undergoes post-translational modifications (PTM), including glycosylation and S1 cleavage. In the secretory pathway, during maturation, a furin-like convertase cleaves full-length Notch at the S1 site, generating a heterodimeric receptor that translocates to the cell surface. At the cell surface, interaction with cognate ligands (Delta and Jagged) on an adjacent cell triggers conformational changes that make the Notch1 heterodimer accessible to ADAM10 metalloprotease for cleavage at the extracellular juxtamembrane S2 site, releasing the ectodomain.^125,126^ This cleavage leaves behind the membrane-bound Notch extracellular truncation (NEXT).^127^ NEXT is then cleaved at the S3 site (between G1743 and V1744 for murine, G1753 and V1754 for human) within the membrane near the cytosolic side by γ-secretase, releasing the Notch intracellular domain (NICD)^43,44^ into the cytosol. The remnant membrane-anchored stub of Notch, known as Nβ, is further cleaved within its TMD by γ-secretase at S4 sites, releasing shorter, secreted Nβ species.^47,128^ The NICD translocates to the nucleus and initiates gene expression after interaction with the DNA-binding transcription factor CSL.^44,129^ Hence, the S3 cleavage by γ-secretase is an essential step in this crucial signaling pathway. The proteolysis of Notch1 by γ-secretase is shown in Fig. 5.

### Other substrates

B cell maturation antigen (BCMA), a cell-surface receptor, is a short γ-secretase substrate that does not need ectodomain shedding before γ-secretase cleavage and is involved in the regulation of plasma cell survival by interaction with its ligands.^130^ Another γ-secretase substrate, Triggering Receptor Expressed on Myeloid Cells 2 (TREM2), is a cell-surface receptor involved in signaling pathways that regulate cytokine secretion and phagocytosis.^131^ The soluble form of TREM2, released after ADAM-like proteolytic cleavage, produces inflammatory cytokines and is involved in the survival of microglia.^132^ Furthermore, many cytokines and other immune receptors, including TNFR1, IL-1R1, IL-1R2, IL6R, and CX3CL1, are also important γ-secretase substrates.^133^

CD44, a γ-secretase substrate, is an adhesion protein that is expressed in most cells and is involved in intracellular signal transduction.^134^ CD44 is physiologically important in hematopoiesis, immune system maintenance, and wound healing, as well as being involved in pathological conditions such as cancer.^134,135^ Another important class of cell–cell adhesion proteins, cadherins, are substrates for γ-secretase.^136,137^ These Ca^2+^-dependent proteins mediate cell adhesion *via* adherens junctions (AJ) and bind to intracellular components, such as catenins, in the cytoplasmic region.^138,139^ Two more classes of proteins that act as synaptic cell-adhesion molecules are pre-synaptic neurexin (NRX) and its primary post-synaptic partner neuroligin (NLG), which are essential in synapse formation and function.^140,141^ Their function at synapses is regulated by γ-secretase processing.^142–145^ γ-Secretase substrates neuregulin (NRG) and its receptor epidermal growth factor receptor (ErbB) are involved in the development and functioning of the nervous system.^146^ γ-Secretase cleavage of cell surface protein family receptor tyrosine kinases (RTKs) generates RTK ICDs, which regulate their signaling pathways. These pathways ultimately activate gene transcription and modulate cell activity. Out of 55 known human RTKs, 27 RTKs have so far been identified as substrates for γ-secretase.^147^

## Substrate processive proteolysis and sequence specificity

### Substrate trimming and the three-pocket model

**Fig. 6:**
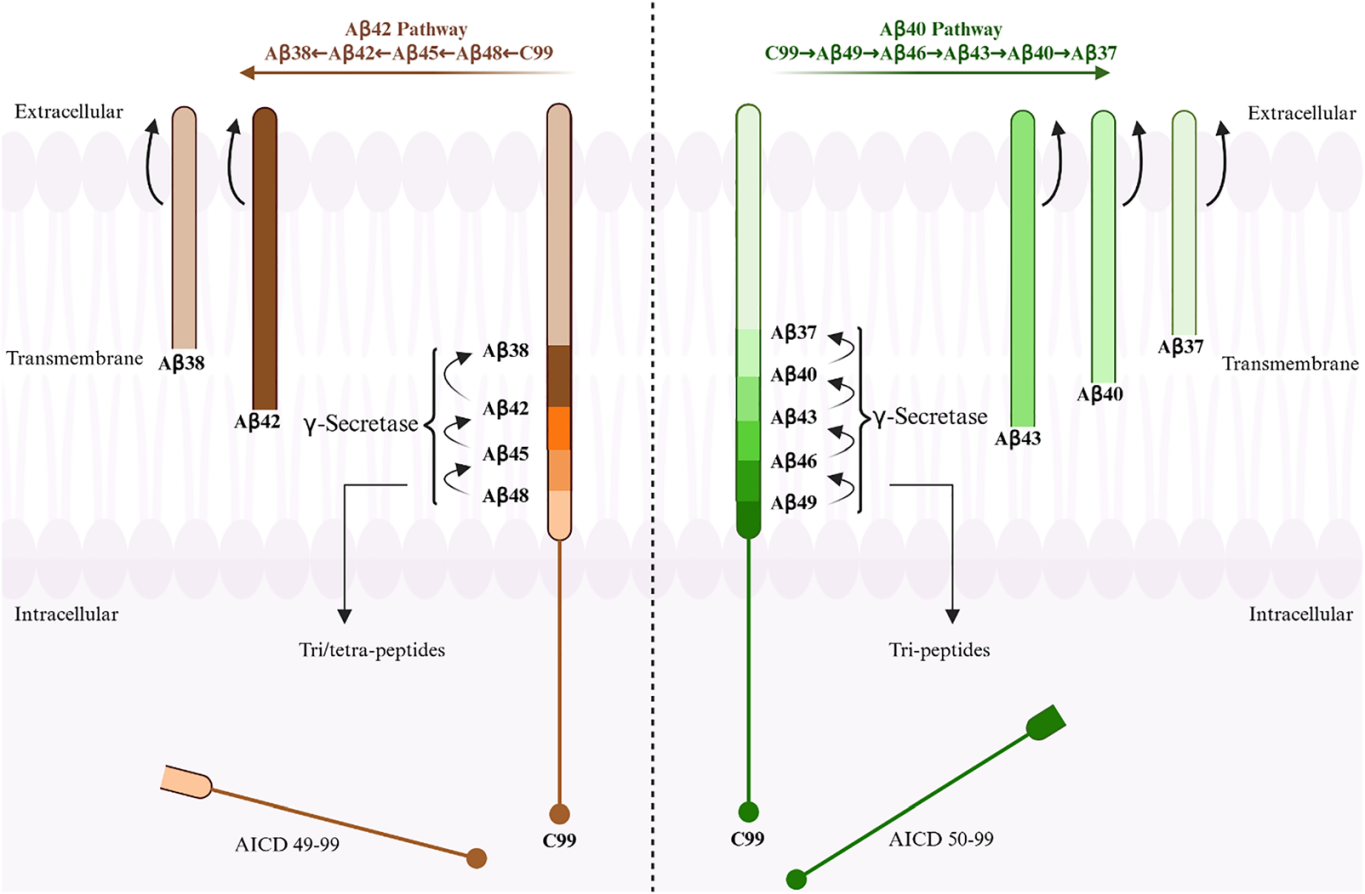
Processive proteolysis of C99 by γ-secretase along the Aβ40 and Aβ42 pathways. Two main pathways that generate Aβ40 (C99 → Aβ49 → Aβ46 → Aβ43 → Aβ40) and/or Aβ42 (C99 → Aβ48 → Aβ45 → Aβ42) and the corresponding AICD species (50–99) and (49–99). The longer Aβ45–49 remains bound to the enzyme in the membrane until further trimmed by the enzyme. The shorter Aβ are released into the extracellular space, while tri-/tetra-peptides are released into the cytoplasm.

Substrate trimming by γ-secretase is discussed in the context of APP processing, which has been extensively studied and is shown in Fig. 6. APP proteolysis by γ-secretase is a complex process.^148^ Initially, APP C99 undergoes endoproteolysis at the ε-site after residues Leu49 or Thr48, generating Aβ49 or Aβ48, respectively, with release of the corresponding APP intracellular domain (AICD), AICD 50–99 or AICD 49–99.^149–151^ Total Aβ and AICD are generated in equimolar proportions.^152^ γ-Secretase then cleaves Aβ49 and Aβ48 in three amino acid increments through its carboxypeptidase activity, producing tripeptide co-products as well as Aβ40 or Aβ42.^105,153–155^ Hence, the two main pathways that generate Aβ40 or Aβ42 are: C99 → Aβ49 → Aβ46 → Aβ43 → Aβ40 and C99 → Aβ48 → Aβ45 → Aβ42, respectively (Fig. 6). Aβ40 can be further trimmed to Aβ37, while Aβ42 is trimmed to Aβ38, generating a tetrapeptide co-product.^155,156^ No clear reason for the generation of the tetrapeptide is known; however, it is thought that the enzyme prefers cleavage between the less crowded G38–V39 bond instead of the more crowded V39–V40 bond to generate Aβ38 and a tetrapeptide.^155^ The longer forms of Aβ peptides (Aβ45–Aβ49) remain bound to the membrane until further processed by γ-secretase and are not found extracellularly.^105^ Additional minor alternative pathways have also been observed, ultimately yielding shorter, secreted forms of Aβ.^157^ Notch1 is very likely processed similarly by the enzyme.^47,128^

**Fig. 7:**
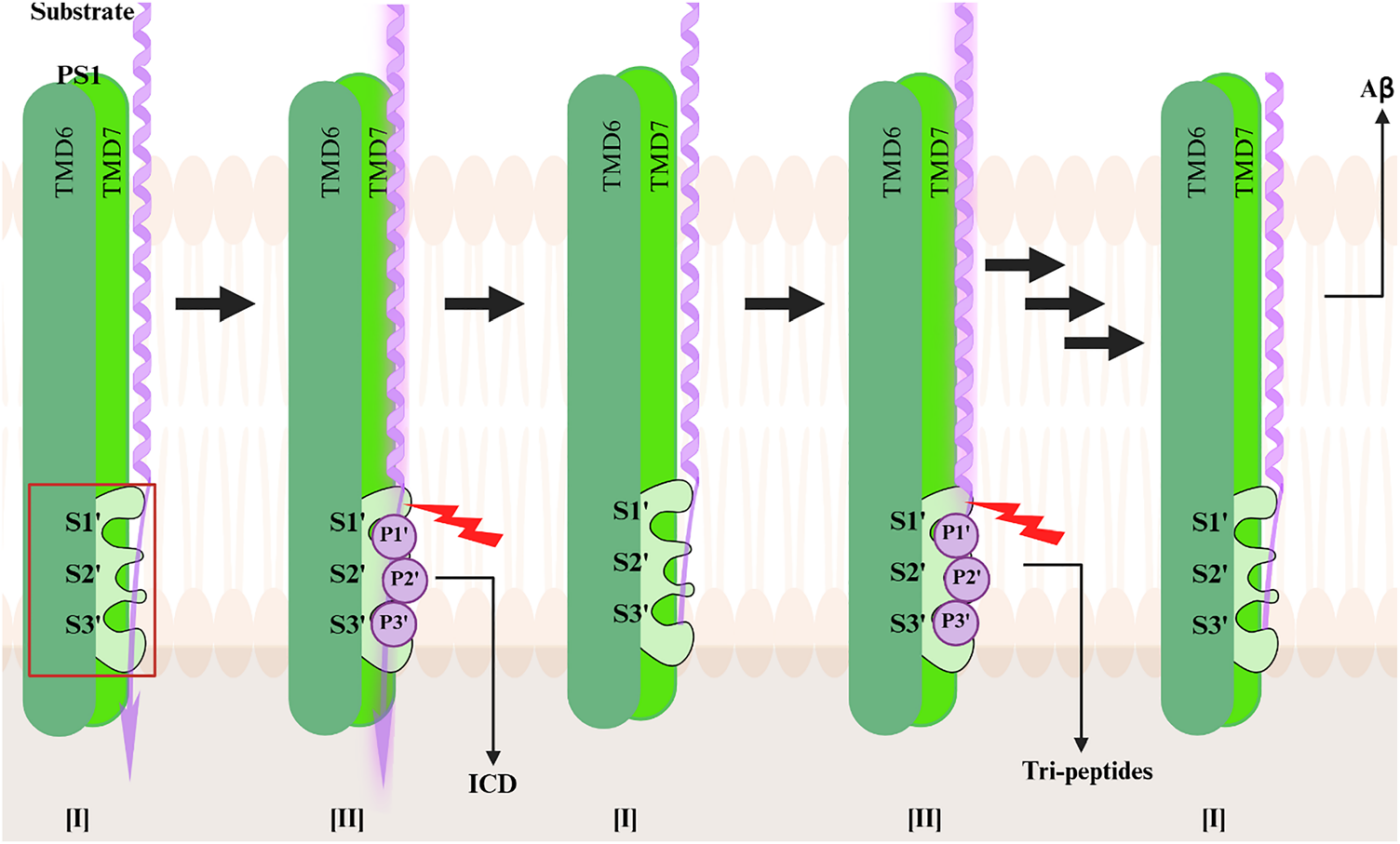
Substrate processing in the three-pocket model of γ-secretase. Three active-site pockets (S1′, S2′ and S3′) of γ-secretase, with S2′ being the smallest. The red rectangle represents the active site of the enzyme. The substrate (purple) enters the active site [I] and unwinds to bind in the active site. The P1′, P2′ and P3′ residues of the substrate occupy the three pockets of the enzyme (purple circles), generating transition state [II], poised for cleavage by the enzyme. The process continues until the tripeptides and Aβs are released intra- and extra-cellularly, respectively.

As early molecular probes for γ-secretase, peptidomimetic transition-state analogue (TSA) inhibitors were initially used to probe the active-site binding pockets of the enzyme.^87^ These probes suggested three pockets in the enzyme active site (S1′, S2′, and S3′) that could accommodate three substrate residues (P1′, P2′ and P3′), as adding a P4′ residue to the TSAs did not change inhibitor potency, and removing the P3′ residues substantially reduced it. These probes further suggested that the S2′ pocket is smaller than the other two pockets.^158,159^ A substrate mutagenesis study further confirmed the “three-pocket model” of the active site, which dictates processive APP carboxypeptidase cleavage to generate tripeptide products.^160^ The three pockets in the active site likely stabilize the unwound substrate and hence its availability for cleavage in a tripeptide pattern (Fig. 7).

### Substrate sequence specificity

**Fig. 8:**
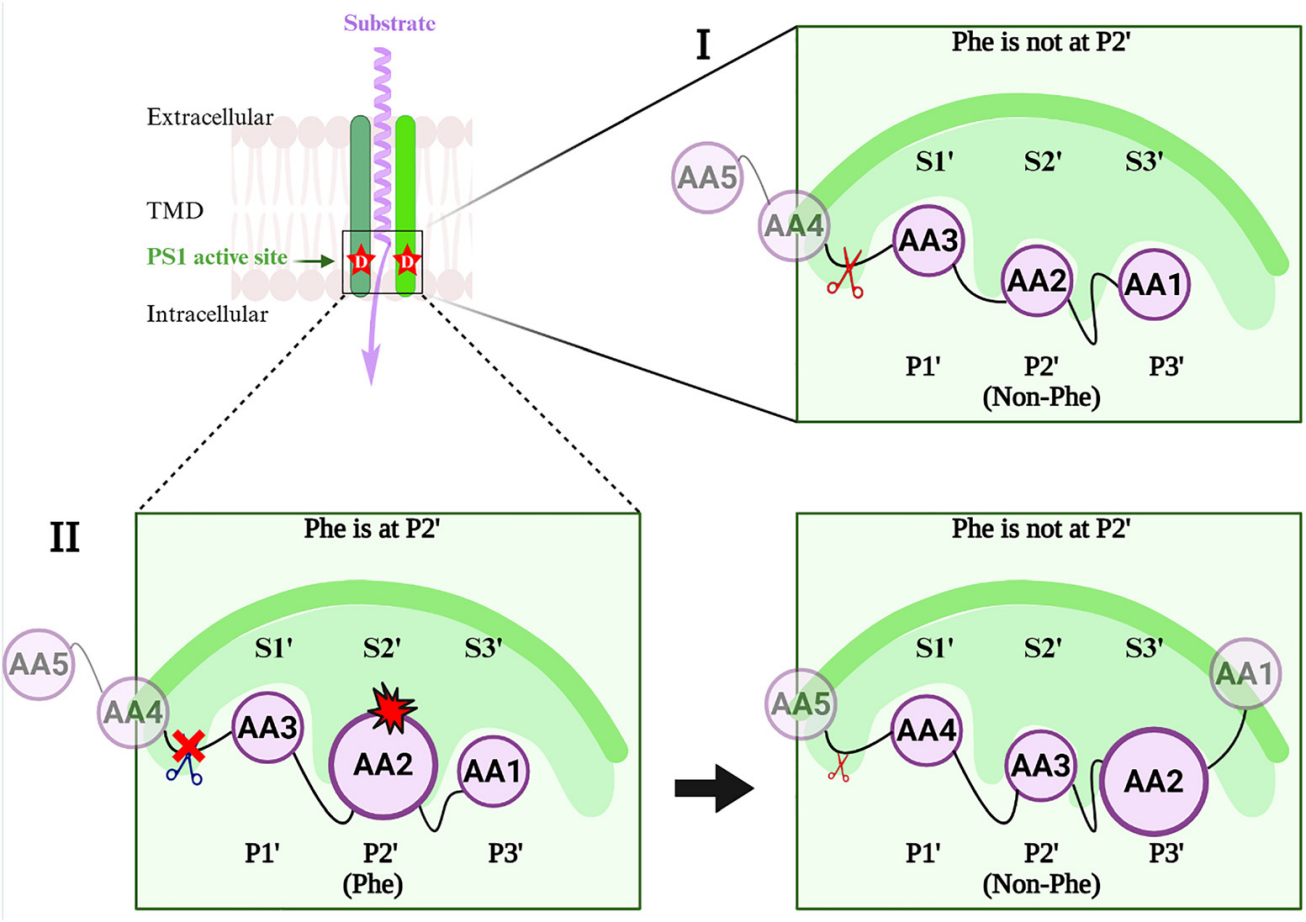
Representation of the phenylalanine specificity rule for substrate cleavage by γ-secretase. The active site of PS1 (3 pockets shown in green) and two different cleavage scenarios of the substrate (purple). (I) Cleavage by γ-secretase (red scissor) occurs between AA3 and AA4, where AA2 is not Phe and hence Phe is not in the P2′ position. Three amino acids in the substrate sequence occupy three active-site pockets of γ-secretase sequentially. (II) Cleavage of the substrate when the P2′ position is occupied with bulkier Phe (AA2). Bulky Phe in the P2′ position clashes (red) with the small S2′ active-site pocket. The substrate hence moves further into the active site of the enzyme, and Phe (AA2) now occupies the bigger S3′ pocket of the active site, and therefore, gets cleaved between AA4 and AA5 to a smaller extent (small red scissors). The scissors represent the peptide bond cleavage site, and AA represents an amino acid in the protein sequence. D represents active site aspartates.

As mentioned earlier, γ-secretase has no sequence specificity for cleavage of its substrates. Analysis of cleavage sites revealed that Val and Leu are favored amino acids for residues P1 (an immediate N-terminal amino acid from the cleavage site) and P1′ (an immediate C-terminal amino acid from the cleavage site), respectively.^161^ The only sequence specificity rule known for γ-secretase cleavage is that bulky amino acids, such as phenylalanine (Phe), are not tolerated at the P2′ position with respect to any cleavage event in APP.^160^ The series of TSA inhibitors with Phe at the P2′ position showed a dramatic loss of potency.^159^ The smaller S2′ pocket in the enzyme active site clashes with the bulkier side chains of Phe and hence cannot bind in the active site for cleavage. However, this rule was established only for APP and its cleavage along both Aβ40- and Aβ42-producing pathways. Very recently, this rule was tested for other γ-secretase substrates such as Notch1, neuregulin-1 (NRG1) and E-Cadherin (CDH1).^128,162^ By installing Phe at the P2′ site respective to ε-cleavage, shifts in the cleavage site were observed, indicating that this rule also applies to these other substrates. Since there are more than 145 substrates for the enzyme, further investigation is needed to determine whether this rule is specific to only a few substrates or is a general phenomenon applicable to a broad range of substrates. This phenylalanine specificity rule is depicted in Fig. 8, where the active site of presenilin is shown. The S2′ pocket is smaller than the other two pockets and cannot accommodate phenylalanine, hence a shift in the cleavage site is observed.

## γ-Secretase in Alzheimer's disease

Alzheimer's Disease (AD) is a devastating neurodegenerative disease that causes cognitive decline and is the most common form of dementia.^163^ Pathophysiological hallmarks include deposition of cerebral plaques composed of Aβ peptides outside neurons and neurofibrillary tangles composed of filamentous tau protein inside neurons. Aggregation of these misfolded proteins, together with other neuropathologies, is widely thought to cause neuronal degeneration.^164^ AD is categorized into two types with varying time of onset: early-onset AD (familial AD, or FAD), and late-onset AD (Sporadic AD, or SAD).^165^ FAD accounts for a very small portion of all AD cases.

Multiple factors, including age, genetics, lifestyle, environmental factors, other diseases, and head injuries, are linked to increased risk of AD. Therefore, various hypotheses, dependent and independent of Aβ, have been proposed and reviewed in detail.^166–168^ However, these hypotheses are not mutually exclusive; some could contribute simultaneously towards the development of AD. All these hypotheses are summarized in Table 1. Based on these various hypotheses, many treatment options for disease management have also been proposed. Despite extensive research, no cure has yet been discovered for the disease. Treatments such as acetylcholinesterase inhibitors (AchEIs) or *N*-methyl d-aspartate (NMDA) receptor antagonists have been used for symptom management and to improve the quality of life of AD patients. Many clinical trials have been done, and many are still in progress for AD based on gene therapy^165^ and amyloid-dependent/independent hypotheses.^167–171^

**Table 1:** Various hypotheses of Alzheimer's disease (AD)

Hypothesis	Explanation
Amyloid cascade hypothesis^172–175^	Aβ42 aggregates to form extracellular Aβ plaques in the brain.
Tau aggregation hypothesis^202–205^	Aggregation of the hyperphosphorylated tau proteins causes neurofibrillary tangles in the brain.
Mitochondrial cascade hypothesis^207,208^	Dysfunctional mitochondria cause an increase in reactive oxygen species, in turn, causing neuronal damage.
Genetic hypothesis^213–217^	ApoE4 allelic variant as a genetic risk factor.
Stalled E-S complex hypothesis^218,219^	FAD-mutant presenilin or APP substrate forms a stable γ-secretase enzyme-substrate complex, triggering synaptic degeneration.
Vascular hypothesis^223–226^	Dysfunctional cerebrovascular system reduces blood supply to the brain.
Infection hypothesis^227,228^	Microbial infection in the brain.
Neurotransmitter hypothesis^166,167^	Imbalance of neurotransmitters, such as acetylcholine and glutamate.
Metal homeostasis hypothesis^166^	Dysregulation of the essential and toxic levels of non-essential metal ions.

### Amyloid hypothesis

**Fig. 9:**
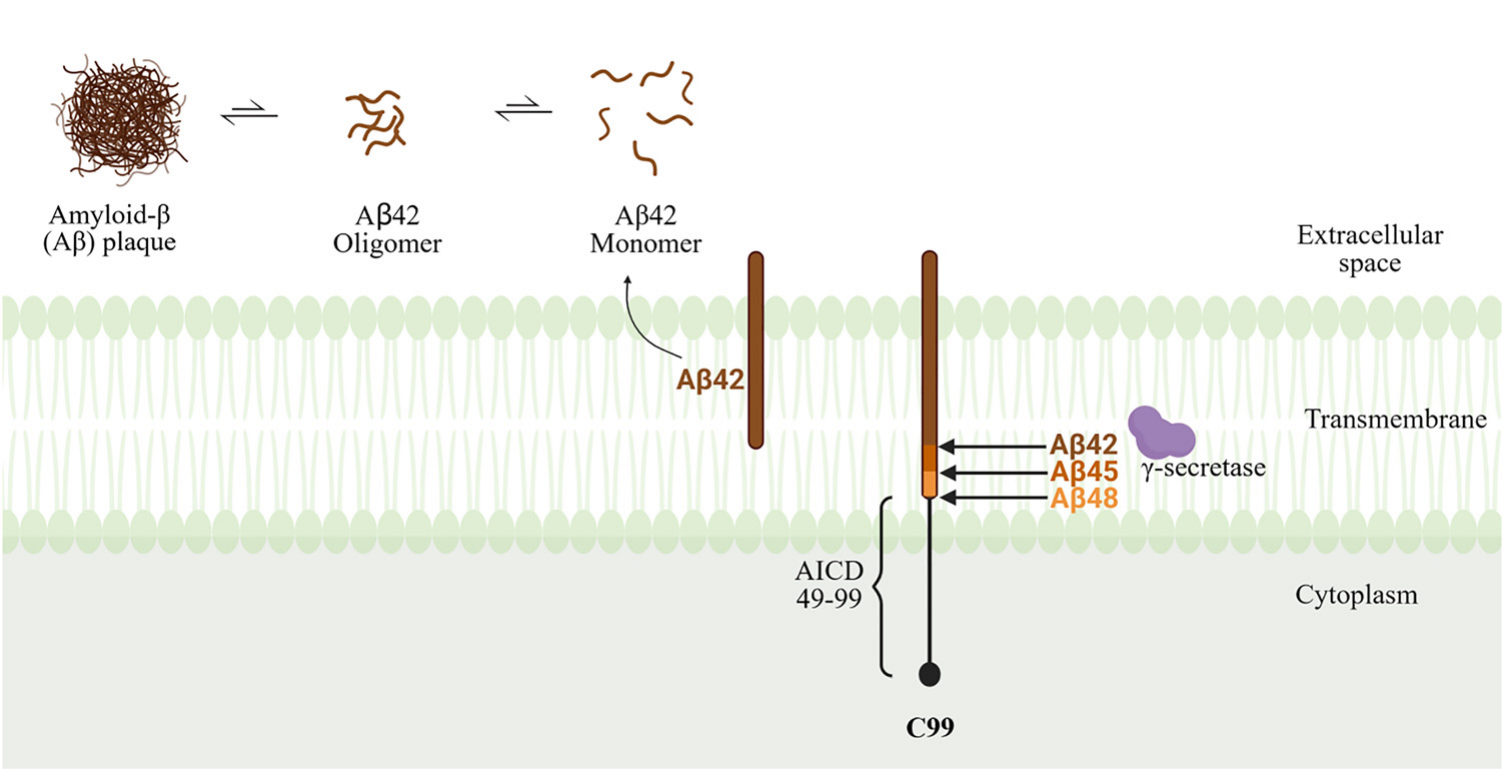
Formation of amyloid β (Aβ) plaques. Generation of Aβ42 from Aβ48 and AICD 49–99 by γ-secretase cleavage. The Aβ42 monomers are released into the extracellular space, which clump together to form oligomers, which aggregate and deposit in the form of Aβ plaques.

The amyloid cascade hypothesis, first proposed in 1991, is the most widely accepted explanation of AD pathogenesis.^172–174^ Its most recent formulation states that soluble Aβ oligomers initiate AD and that other pathological features result from Aβ aggregation.^175^ As a corollary, symptoms of AD were predicted to be improved with reduction in Aβ and plaques level,^176^ which mainly consists of Aβ42.^177,178^ Dominant missense mutations in APP (generally found in and around the small Aβ region of APP) and in PS1 and PS2 were discovered to be associated with FAD, and these mutations were soon found to increase the proportion of aggregation-prone Aβ42.^179–182^ In contrast, SAD appears to involve the failure to clear generated Aβ, leading to increased Aβ42 deposition in the brain, as evidenced in human studies.^175,183,184^ In either case, increased cerebral Aβ42 levels lead to oligomerization of Aβ42 and deposition as plaques, with the oligomers implicated in synaptic dysfunction and the plaques thought to serve as a reservoir for oligomers (Fig. 9).^185^ Synaptic dysfunction is followed by synaptic loss, neurodegeneration, and ultimately dementia.^175^

The amyloid cascade hypothesis was further supported by the identification of a missense APP mutant (A673T) near the β-secretase cleavage site, which decreases cleavage at this site and provides protection against AD and age-related cognitive decline.^186^ Aβ oligomers have been reported to elicit different pathways to impart neuronal toxicity in AD.^166^ Based on the amyloid hypothesis, many clinical trials targeting Aβ have been conducted; however, most (such as BACE and γ-secretase inhibitors) were unsuccessful because of the severe side effects.^187,188^ Another reason for the failure of anti-amyloid trials is that the treatments are not provided early during the disease progression. A clinical study in dominantly inherited AD patients had shown that AD biomarkers, such as a reduction of Aβ42 and an increase of tau protein in CSF (cerebrospinal fluid), as well as deposition of Aβ in plaques, appear decades before the onset of AD symptoms.^189^ The recent development of passive anti-amyloid immunotherapies, however, has shown promise.^190,191^ The clinical trials of anti-amyloid monoclonal antibodies (MABs) have shown that MAB treatments cleared plaques and decreased the rate of cognitive decline, albeit modestly, providing further support for the amyloid hypothesis and the idea that amyloid is a viable target for AD treatment.^192,193^ Recently, aducanumab, lecanemab, and donanemab were approved by the FDA^194,195^ as disease-modifying therapies (DMTs) for the treatment of early AD and mild cognitive impairment (MCI),^192^ and many are currently under development.^196^ In addition to developing DMTs for symptomatic AD patients, the new Aβ targeted trials, including MABs, are also focusing on primary and secondary prevention in asymptomatic and presymptomatic high-risk individuals carrying genetic mutations.^197^ A recent clinical study of long-term gantenerumab treatment in high-risk dominantly inherited AD (DIAD) individuals has shown potential in delaying the onset of AD symptoms.^198^ However, recent *in vitro* studies on the effects of FAD mutations in APP and PS1 revealed that not all mutations increase the Aβ42/Aβ40 ratio.^199,200^ Although Aβ appears to be centrally involved in AD, therapies that target only Aβ may be insufficient.^201^

### Amyloid-independent hypotheses

Despite huge research efforts focused on the amyloid hypothesis, the failure to find effective Aβ-targeting therapeutics has led to consideration of other hypotheses and the formulation of alternative therapeutic strategies. In contrast to the amyloid cascade hypothesis, many of these alternative hypotheses posit that amyloid plaque deposition in the AD brain is a consequence, not the cause, of other pathological changes.

The tau aggregation hypothesis of AD is perhaps the second-most accepted, as deposition of tau-containing neurofibrillary tangles (NFTs) is one of the hallmark pathological features of AD. This hypothesis is based on the accumulation of NFTs generated from the aggregation of hyperphosphorylated tau proteins. Tau proteins have different isoforms, resulting from alternative splicing, and are essential components that provide microtubule stability and integrity.^202,203^ Although different pathologies, amyloid and tau aggregations may work together towards AD pathogenesis and progression.^166^ Aberrant phosphatase and kinase activity lead to tau hyperphosphorylation, which, in turn, aggregates and deposits as NFT.^202,204^ Abnormal tau truncation, through proteolysis, is another cause of tau aggregation.^205^ Additionally, other tau modifications, such as acetylation, nitration, and glycosylation, could also contribute to tau aggregation.^205^ Furthermore, pathological tau aggregates can apparently spread from neuron to neuron *via* a seeding mechanism, leading to spread throughout the brain and associated neuronal loss.^203^

Mitochondria act as the ‘powerhouse’ of the cell, supplying energy in the form of adenosine triphosphate (ATP) through oxidative phosphorylation. Efficient functioning of mitochondria is particularly essential in neurons, as they have high energy requirements.^206^ The mitochondrial cascade hypothesis, first proposed in 2004,^207^ suggests that mitochondrial dysfunction and resulting increase in reactive oxygen species (ROS) cause neuronal damage, hence contributing to AD pathogenesis. Mitochondrial dysfunction—involving dysregulation of mitochondrial fusion and fission, trafficking, and mitophagy—is observed in the AD brain.^206^ Mitochondrial dysfunction is reported to affect APP expression, processing and amyloid deposition, in addition to affecting tau phosphorylation, inflammation and oxidative stress.^208^ Oxidative stress occurs from disrupted redox systems, where an imbalance between biological oxidants and antioxidants is observed,^209^ and involves increased production of ROS or other reactive species, which causes deterioration of neuronal cells.^210^ In AD, the activity of mitochondrial enzymes in the oxidative pathway is altered,^211^ and the number of mitochondria is reduced. Moreover, abnormal mitochondrial DNA is associated with AD.^212^

Besides the FAD mutations found in APP and PS, allelic variation of apolipoprotein E (ApoE) is a major genetic risk factor for AD. The ApoE protein acts as a lipid carrier and is involved in lipid metabolism.^213^ Of the three ApoE alleles found in humans, ε2 (ApoE2), ε3 (ApoE3) and ε4 (ApoE4), with differences only at amino acids 112 and 158, ε4 is strongly associated with AD.^213–215^ ApoE4 is associated with reduced Aβ clearance in the brain,^215^ increased hyperphosphorylation of tau^216^ and stabilization of Aβ oligomers.^214^ Additionally, variants, such as R47H in the microglial transmembrane protein TREM2, are reported to be another genetic risk factor contributing to AD development.^217^

Recently, the “stalled enzyme–substrate (E–S) complex” hypothesis was proposed for FAD, which posits that FAD mutations in either presenilin (the catalytic component of γ-secretase) or APP (one of its substrates) may lead to stabilized E–S complexes that trigger synaptic degeneration. These stalled E–S complexes lead to deficient processing of substrates, producing increased proportions of long Aβ peptides in the case of the APP C99 substrate, thereby generally increasing the Aβ42/Aβ40 ratio. A *C. elegans* model system for FAD was developed and leveraged to show that FAD mutations trigger synaptic degeneration either through deficient processing of other essential substrates (*i.e.*, loss of function) or through the stalled E–S complex *per se* (*i.e.* gain of toxic function).^218,219^ However, a dominant loss-of-function mechanism alone can be ruled out, as PS1 mutants in the human population that lead to nonsense-mediated decay of the mRNA (and therefore haploinsufficiency) cause a hereditary skin disease, not neurodegeneration.^220^ Moreover, in the *C. elegans* model system, transgenic lines expressing catalytically dead PS1 (D257A) do not display a neurodegenerative phenotype, as seen with FAD mutations.^221^ The *C. elegans* system further revealed that APP C99 mutations that block Aβ production, and comparable Notch “N99” mutations that reduce ε cleavage, likewise trigger synaptic degeneration, suggesting that stalled substrates other than APP can be neurotoxic. Consistent with these findings, a mouse knock-in model with an FAD-mutant PS1 (L435F) was recently found to develop age-dependent neurodegeneration even when the APP gene was knocked out.^222^

The vascular hypothesis suggests that the dysfunctional cerebrovascular system may synergistically contribute to the development of AD.^223^ Clinical studies suggest that cerebral vascular dysfunction and reduced blood flow occur before the appearance of hallmarks of AD.^224,225^ Vascular diseases, such as hypertension, diabetes, hyperlipidemia, and hypercholesterolemia, have been linked with the occurrence and progression of AD in mouse and clinical studies.^226^ These diseases cause damage in the cerebral vasculature, which leads to brain dysfunction and neurodegeneration in AD.

Another hypothesis connects microbes, microbial infection and neuroinflammation to sporadic AD pathogenesis.^227,228^ Considerable evidence suggests that Aβ peptides have antimicrobial activity^229–231^ and their synthesis is increased in microbial infections.^227^ Age-dependent altered gut microbiota is also connected to dementia^232^ as well as to increased permeability and dysfunction of the blood–brain barrier (BBB),^233^ and hence with the onset of AD.

Some older hypotheses propose an association of an imbalance of neurotransmitters, such as acetylcholine or glutamate, as well as calcium homeostasis, with the development of AD.^166,167^ The cholinergic hypothesis links decreased synaptic acetylcholine—a neurotransmitter involved in memory and cognitive functions—with AD, while the glutamatergic hypothesis links overactivity of ionotropic NMDA-type glutamine receptors to AD. In addition to calcium, dysregulation of other essential metal ions, such as zinc, iron, and copper, as well as toxic intake of non-essential metal ions, such as aluminum, lead, and cadmium, are reported to be associated with AD pathology.^166^ Other than these, amyloid cross-seeding, lymphatic system, microRNA, ion channel, cell cycle, autoimmune, granuloma, dysregulated Reelin homeostasis, and pesticide-induced neuropathology hypotheses have also been proposed.^166,167,234–236^ Many recent reviews propose integration of multiple hypotheses and their concurrent and interdependent occurrence.^237–239^

## The role of γ-secretase in biology and diseases other than AD

**Fig. 10:**
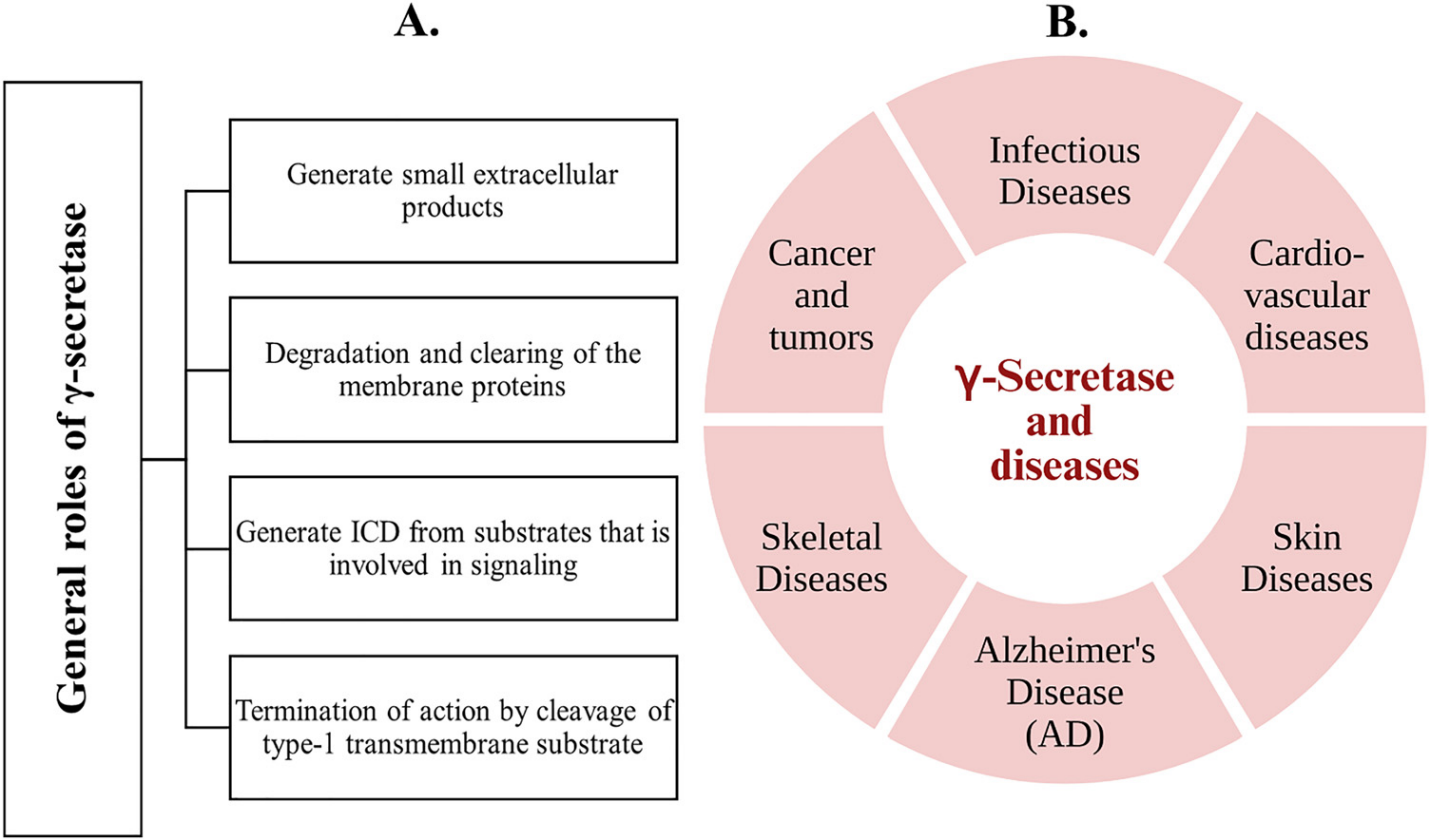
General roles of γ-secretase in biology (A) and disease (B).

Normal γ-secretase functioning is essential during embryonic development as well as adult well-being. As mentioned, γ-secretase has many type I integral membrane substrates besides APP. Given the wide range of substrates, the enzyme is involved in a broad range of biological functions (Fig. 10).^68,240^ γ-Secretase-mediated pathways are associated with various biological systems (*e.g.*, nervous, cardiovascular, skin, kidney, and immune), and hence γ-secretase is involved in many diseases related to these systems as well as cancers (Fig. 10).^240^ The functions of γ-secretase can generally be categorized into degradation and clearing of membrane proteins, extracellular release of shorter peptides from substrates, cell signaling by released ICDs, or termination of action of full-length substrates, depending upon the substrate cleaved (Fig. 10). For example, it was suggested recently that γ-secretase not only generates physiologically active fragments from substrates but also inactivates neurotoxic C99.^118^

The membrane-associated C-terminal stubs that remain after ectodomain shedding require clearance from the membrane; γ-secretase is proposed to perform this critical degradation function, hence acting as the ‘proteasome of the membrane’.^107^ This cleavage generates secreted N-terminal peptides as well as C-terminal intracellular domains (ICDs), which may themselves serve some biological roles. The AICD, generated after ε-cleavage of APP by γ-secretase, has been proposed to have many roles, including transcription regulation,^241,242^ but these remain controversial. The NICD generated by Notch family receptor cleavage is involved in gene transcription, as noted earlier. Besides Notch, ICDs from alcadeins, cadherins, Notch ligands Delta and Jagged, CD44, and many other substrates have also been reported to translocate to the nucleus and affect transcription.^68,101^ Furthermore, alcadein-α ICD is involved in regulating the trafficking of membrane proteins, specifically APP.^243^

γ-Secretase is also known to be critically involved in regulating inflammatory responses and innate immunity.^244^ For example, γ-secretase is involved in an adipocyte-mediated inflammatory signaling pathway and regulation of adipose tissue inflammation, likely *via* IL-6.^245^ γ-Secretase also cleaves cell-adhesion proteins to regulate the assembly or disassembly of adhesion junctions. For example, cleavage of E-cadherin by γ-secretase promotes dissociation of adherens junctions.^136^ γ-Secretase is found in pre- as well as post-synapses, where it is involved in synaptic transmission and autoregulation^246^ as well as helping to maintain synaptic plasticity.^247^ Altered γ-secretase processing of neuregulin-1 (NRG-1) is strongly linked with an increased risk of schizophrenia.^248^

Dysregulated Notch signaling is implicated in cancers of various biological systems, including nervous, digestive, respiratory, blood, reproductive, and urinary systems.^124^ The CD-44-ICD was shown to possess oncogenic cell transformation activity affecting tumorigenesis.^249^ Protein-tyrosine kinase 7 (PTK7) is a regulator of planar cell polarity and is upregulated in many cancers. Importantly, the ICD generated from its γ-secretase cleavage translocates to the nucleus and enhances tumorigenesis.^250^ The process of angiogenesis is physiologically essential for development and tissue repair, but pathological angiogenesis is involved in diseases such as cancer. γ-Secretase has been reported to play a key role in regulating angiogenesis through various factors, such as Notch, ErbB4, insulin-like growth factor-1 receptor (IGF1-R), vascular endothelial growth factor receptor-1 (VEGFR-1), cadherin, and APP.^251^

γ-Secretase is also involved in cardiac development as well as maintenance and regulation of cardiac functions *via* many of its substrates, such as Notch1, the NRG1-ErbB4 pathway, and subunits of voltage-gated sodium and potassium channels.^252^ Normal γ-secretase functioning is essential for maintaining cardiac health, as various cardiac disorders were observed in γ-secretase knockout animal models.^252^ γ-Secretase also plays a role in metabolism and metabolic diseases *via* Notch signaling.^253^ Interestingly, hepatic γ-secretase regulates cleavage of the low-density lipoprotein receptor (LDLR) and affects VLDL/LDL uptake in a Notch-independent manner.^254^ Normal Notch signaling, and hence γ-secretase activity, is physiologically essential in bone biology, and both Notch overexpression and loss of Notch activity are involved in bone pathology in mice.^255^ Notch plays a critical role during skeletal development by regulating the differentiation and function of osteoblast and osteoclast cells.^256,257^

γ-Secretase also plays a vital role in skin biology, and its inhibition can cause skin abnormalities.^220,258^ γ-Secretase likely acts through various pathways in skin biology, including Notch, EGFR and phosphoinositide-3-kinase (PI3K).^259^ About 57 genetic mutations in γ-secretase components PS1, NCT, and PEN-2 have been reported to date that cause a debilitating inflammatory skin disease called acne inversa (AI) or hidradenitis suppurativa (HS).^260^

γ-Secretase is also proposed to be involved in infectious diseases. The proteolytic activity of γ-secretase was reported to be required for efficient human cytomegalovirus (HCMV) replication at the transcriptional level, independent of Notch signaling.^261^ Interestingly, for human papillomavirus (HPV) infections, although γ-secretase is necessary, its proteolytic action is not.^262^ In HPV infections, γ-secretase serves a chaperone function and promotes insertion of L2 capsid protein in endosomal membranes at low pH.^262^

In addition to their role in the γ-secretase complex and enzymatic activity, the individual γ-secretase components are reported to have other non-proteolytic roles in biology.^263^ For instance, PS can act as a scaffolding protein in mammals as well as plants and amoeba.^264^ PS is associated with other roles as well, such as calcium homeostasis, autophagy, protein trafficking/degradation, apoptosis, inflammation, and synaptic functions.^263,265^ PS mutations are also reportedly involved in neurodegenerative diseases other than AD, such as frontotemporal dementia.^266^ APH1 and PEN2 possess protective anti-apoptotic activity *via* the p53 pathway, which is independent of γ-secretase activity.^267^ NCT was also found to be cell-protective by controlling cell-death *via* PI3K/Akt- and p53-dependent pathways, independently of γ-secretase activity.^268^

## γ-Secretase as a therapeutic target

γ-Secretase, first discovered in the context of AD, has been a major therapeutic target for AD for three decades. As Aβ plaques are a pathological hallmark of AD, and FAD mutations are found in APP and PS, the development of γ-secretase inhibitors (GSI) was of keen interest. Inhibition of γ-secretase activity with a GSI to reduce Aβ production was first demonstrated *in vivo* with the dipeptide analog DAPT.^269^ Many other GSIs have been developed since then. Sulfonamides (*e.g.* BMS299897),^270^ benzodiazepines (LY-411575),^271^ and benzolactams (LY-450139)^272^ were subsequently developed that showed reductions in brain Aβ production. However, GSIs also showed concerning toxicities upon chronic treatment, similar to phenotypes observed upon knockout of *Notch1* or *PS1*, suggesting that GSI toxicities are due to inhibition of Notch cleavage and signaling.^271^

In late-stage clinical trials of GSIs semagacestat and avagacestat, adverse effects such as skin cancer and immunosuppression were observed.^273,274^ In addition to blocking cleavage of APP, these GSIs also block γ-secretase-mediated Notch cleavage,^275^ which is likely the reason for these adverse reactions. Therefore, the development of GSIs with selectivity towards APP *vs.* Notch was thought to be a necessity for their therapeutic usefulness as AD treatments. More concerning, however, treatment with GSIs led to cognitive worsening and AD progression.^273,276^ Furthermore, the clinical failure of GSIs due to worsening of cognitive symptoms correlates with impaired clearance of C99. The resulting increase in C99 accumulation may aggravate disease conditions, suggesting, therefore, that γ-secretase inhibition is not a good treatment strategy for AD.^118^

Despite their clinical failure as treatments for AD, GSIs are being repurposed to treat many other diseases. γ-Secretase is proposed to be a good therapeutic target for treating excessive angiogenesis; however, the development of molecules specific to the substrates involved in this process is needed.^251^ Liver-specific GSIs might be effective for the treatment of various metabolic diseases characterized by hypertriglyceridemia.^254^ GSIs reduce LDLR cleavage, hence stabilizing it and reducing levels of triglyceride-rich lipoproteins in the plasma.^254^

Inhibition of Notch signaling with GSIs has also been reported to improve glucose tolerance and insulin sensitivity, while reducing hepatic glucose production. Hence, GSIs may be a beneficial treatment option for diabetes.^277^ Recently, liver-targeted GSI-nanoparticle treatment was shown to provide localized hepatic Notch-signaling inhibition, which led to reduced intestinal side-effects with improved obesity-induced glucose tolerance and reduced liver fibrosis in mice.^278^ Controlling Notch signaling by downregulation using GSIs could be a useful strategy in certain bone diseases;^255^ however, it comes with risks of off-target effects.^256^ Furthermore, transient treatment with the dipeptide analog GSI DAPT was shown to enhance bone formation and fracture repair *via* Notch pathway inhibition in mice, suggesting therapeutic use of GSI in treating skeletal fractures.^279^

Given that certain γ-secretase substrates, especially Notch, are involved in tumorigenesis, many GSIs have also been tested as anti-cancer agents with promising pre-clinical trials, but without much clinical success.^280,281^ Recently, the GSI nirogacestat was shown to be beneficial for desmoid tumors in clinical trials^282^ and has been approved by the FDA as a first targeted therapy for this condition. GSI treatment has been shown to improve the efficacy of BCMA-targeted CAR-T (chimeric antigen receptor-T) cell therapy in multiple myeloma.^283^ GSIs were also shown to inhibit HCMV viral replication and hence are potential anti-HCMV agents.^261^

γ-Secretase modulators (GSMs), on the other hand, could be a safer option to target γ-secretase in AD.^284^ GSMs do not bind in the active site, but instead bind to an allosteric site on the enzyme to modulate γ-secretase activity, which lowers Aβ42 levels without affecting total Aβ levels (*i.e.*, they are not inhibitors). Specifically, GSMs reduce pathological Aβ42 levels by increasing the conversion of Aβ42 to Aβ38.^286–288^ Importantly, GSMs do not affect the proteolysis and signaling of Notch^284^ or other substrates.^289^ The first GSMs identified were a subset of non-steroidal anti-inflammatory drugs (NSAIDs).

These first-generation GSMs had low potency. Second-generation GSMs, such as NSAID-based aryl acetic acids, non-NSAID heterocycles, and natural product analogs, apparently have different mechanisms of action and Aβ-altering profiles.^285,290^ The heterocyclic GSMs include arylimidazoles (*e.g.* E2012, E2212), oxadiazolines and oxadiazines, pyridines, pyrimidines, morpholines and many more.^285^ A lead compound from the newly discovered triazolo-azepine class of GSMs has shown good pharmacokinetic, toxicological and pharmacodynamic properties and has potential for further advancements.^291^ A recent report mentions the development of a new pyridazine-based GSM with promising preclinical data and potential for early clinical trials.^292^ Another recent report identified GSMs that could be effective not only for sporadic AD but also for FAD, including the aggressive PS1 L166P mutant, which has been resistant to most GSMs.^293^

Although GSMs have potential for development as anti-AD treatments, the efficacy of the GSMs or any other molecules targeting Aβ42 in slowing disease progression in human trials still needs to be demonstrated.^148^ Targeting Aβ or γ-secretase alone may not be sufficient for treating AD, but combination therapies targeting multiple pathologies and factors at once might be effective.^294^

## Conclusion

Since its discovery, γ-secretase has been of constant interest to the scientific community. It is the most complicated and promiscuous intramembrane proteolytic enzyme, known for its wide range of substrates. Hence, the enzyme plays essential roles in biology and is involved in a myriad of biological functions. Given its importance in human disease, the enzyme has also been a target for the development of therapeutics for many pathological conditions for decades, and many are currently in clinical trials. Our understanding since the first description of γ-secretase activity has improved dramatically with biochemical, synthetic and structural approaches. Nevertheless, many uncovered aspects remain to be revealed through extensive research to gain a full understanding of the enzyme and its potential as a successful clinical target.

## Conflicts of interest

There are no conflicts of interest to declare.

## Data Availability

This is a review article. Data availability is not applicable.

## References

[cit1] Wolfe M. S., Kopan R. (2004). Intramembrane Proteolysis: Theme and Variations. Science.

[cit2] Kamp F., Winkler E., Trambauer J., Ebke A., Fluhrer R., Steiner H. (2015). Intramembrane Proteolysis of β-Amyloid Precursor Protein by γ-Secretase Is an Unusually Slow Process. Biophys. J..

[cit3] Langosch D., Steiner H. (2017). Substrate processing in intramembrane proteolysis by γ-secretase – the role of protein dynamics. Biol. Chem..

[cit4] Dickey S. W., Baker R. P., Cho S., Urban S. (2013). Proteolysis inside the Membrane Is a Rate-Governed Reaction Not Driven by Substrate Affinity. Cell.

[cit5] Arutyunova E., Panwar P., Skiba P. M., Gale N., Mak M. W., Lemieux M. J. (2014). Allosteric regulation of rhomboid intramembrane proteolysis. EMBO J..

[cit6] Brown M. S., Ye J., Rawson R. B., Goldstein J. L. (2000). Regulated Intramembrane Proteolysis. Cell.

[cit7] Ha Y. (2009). Structure and mechanism of intramembrane protease. Semin. Cell Dev. Biol..

[cit8] Sannerud R., Annaert W. (2009). Trafficking, a key player in regulated intramembrane proteolysis. Semin. Cell Dev. Biol..

[cit9] Erez E., Fass D., Bibi E. (2009). How intramembrane proteases bury hydrolytic reactions in the membrane. Nature.

[cit10] Wolfe M. S. (2009). Intramembrane-cleaving Proteases. J. Biol. Chem..

[cit11] Urban S. (2013). Mechanisms and cellular functions of intramembrane proteases. Biochim. Biophys. Acta, Biomembr..

[cit12] Strisovsky K. (2013). Structural and mechanistic principles of intramembrane proteolysis – lessons from rhomboids. FEBS J..

[cit13] Rawson R. B., Zelenski N. G., Nijhawan D., Ye J., Sakai J., Hasan M. T., Chang T. Y., Brown M. S., Goldstein J. L. (1997). Complementation Cloning of, a Gene Encoding a Putative Metalloprotease Required for Intramembrane Cleavage of SREBPs. Mol. Cell.

[cit14] Urban S., Lee J. R., Freeman M. (2001). Drosophila Rhomboid-1 Defines a Family of Putative Intramembrane Serine Proteases. Cell.

[cit15] Kühnle N., Dederer V., Lemberg M. K. (2019). Intramembrane proteolysis at a glance: From signalling to protein degradation. J. Cell Sci..

[cit16] Freeman M. (2014). The Rhomboid-Like Superfamily: Molecular Mechanisms and Biological Roles. Annu. Rev. Cell Dev. Biol..

[cit17] Manolaridis I., Kulkarni K., Dodd R. B., Ogasawara S., Zhang Z., Bineva G., O’Reilly N., Hanrahan S. J., Thompson A. J., Cronin N., Iwata S., Barford D. (2013). Mechanism of farnesylated CAAX protein processing by the intramembrane protease Rce1. Nature.

[cit18] Kroos L., Akiyama Y. (2013). Biochemical and structural insights into intramembrane metalloprotease mechanisms. Biochim. Biophys. Acta, Biomembr..

[cit19] Wolfe M. S. (2006). The γ-Secretase Complex: Membrane-Embedded Proteolytic Ensemble. Biochemistry.

[cit20] Wolfe M. S. (2020). Unraveling the complexity of γ-secretase. Semin. Cell Dev. Biol..

[cit21] Selkoe D. J. (1994). Cell Biology of the Amyloid beta-Protein Precursor and the Mechanism of Alzheimer's Disease. Annu. Rev. Cell Biol..

[cit22] Haass C., Selkoe D. J. (1993). Cellular processing of β-amyloid precursor protein and the genesis of amyloid β-peptide. Cell.

[cit23] De Strooper B. (2003). Aph-1, Pen-2, and Nicastrin with Presenilin Generate an Active γ-Secretase Complex. Neuron.

[cit24] Chartier-Harlin M.-C., Crawford F., Houlden H., Warren A., Hughes D., Fidani L., Goate A., Rossor M., Roques P., Hardy J., Mullan M. (1991). Early-onset Alzheimer's disease caused by mutations at codon 717 of the β-amyloid precursor protein gene. Nature.

[cit25] Goate A., Chartier-Harlin M.-C., Mullan M., Brown J., Crawford F., Fidani L., Giuffra L., Haynes A., Irving N., James L., Mant R., Newton P., Rooke K., Roques P., Talbot C., Pericak-Vance M., Roses A., Williamson R., Rossor M. (1991). *et al.*, Segregation of a missense mutation in the amyloid precursor protein gene with familial Alzheimer's disease. Nature.

[cit26] Rogaev E. I., Sherrington R., Rogaeva E. A., Levesque G., Ikeda M., Liang Y., Chi H., Lin C., Holman K., Tsuda T., Mar L., Sorbi S., Nacmias B., Piacentini S., Amaducci L., Chumakov I., Cohen D., Lannfelt L., Fraser P. E. (1995). *et al.*, Familial Alzheimer's disease in kindreds with missense mutations in a gene on chromosome 1 related to the Alzheimer's disease type 3 gene. Nature.

[cit27] Sherrington R., Rogaev E. I., Liang Y., Rogaeva E. A., Levesque G., Ikeda M., Chi H., Lin C., Li G., Holman K., Tsuda T., Mar L., Foncin J.-F., Bruni A. C., Montesi M. P., Sorbi S., Rainero I., Pinessi L., Nee L. (1995). *et al.*, Cloning of a gene bearing missense mutations in early-onset familial Alzheimer's disease. Nature.

[cit28] Duff K., Eckman C., Zehr C., Yu X., Prada C.-M., Perez-tur J., Hutton M., Buee L., Harigaya Y., Yager D., Morgan D., Gordon M. N., Holcomb L., Refolo L., Zenk B., Hardy J., Younkin S. (1996). Increased amyloid-β42(43) in brains of mice expressing mutant presenilin 1. Nature.

[cit29] Lemere C. A., Lopera F., Kosik K. S., Lendon C. L., Ossa J., Saido T. C., Yamaguchi H., Ruiz A., Martinez A., Madrigal L., Hincapie L., Arango L. J. C., Anthony D. C., Koo E. H., Goate A. M., Selkoe D. J., Arango V. J. C. (1996). The E280A presenilin 1 Alzheimer mutation produces increased Aβ42 deposition and severe cerebellar pathology. Nat. Med..

[cit30] De Strooper B., Saftig P., Craessaerts K., Vanderstichele H., Guhde G., Annaert W., Von Figura K., Van Leuven F. (1998). Deficiency of presenilin-1 inhibits the normal cleavage of amyloid precursor protein. Nature.

[cit31] Herreman A., Serneels L., Annaert W., Collen D., Schoonjans L., De Strooper B. (2000). Total inactivation of γ–secretase activity in presenilin-deficient embryonic stem cells. Nat. Cell Biol..

[cit32] Zhang Z., Nadeau P., Song W., Donoviel D., Yuan M., Bernstein A., Yankner B. A. (2000). Presenilins are required for γ-secretase cleavage of β-APP and transmembrane cleavage of Notch-1. Nat. Cell Biol..

[cit33] Borchelt D. R., Thinakaran G., Eckman C. B., Lee M. K., Davenport F., Ratovitsky T., Prada C.-M., Kim G., Seekins S., Yager D., Slunt H. H., Wang R., Seeger M., Levey A. I., Gandy S. E., Copeland N. G., Jenkins N. A., Price D. L., Younkin S. G., Sisodia S. S. (1996). Familial Alzheimer's Disease–Linked Presenilin 1 Variants Elevate Aβ1–42/1–40 Ratio *In Vitro* and *In Vivo*. Neuron.

[cit34] Citron M., Westaway D., Xia W., Carlson G., Diehl T., Levesque G., Johnson-wood K., Lee M., Seubert P., Davis A., Kholodenko D., Motter R., Sherrington R., Perry B., Yao H., Strome R., Lieberburg I., Rommens J., Kim S. (1997). *et al.*, Mutant presenilins of Alzheimer's disease increase production of 42-residue amyloid β-protein in both transfected cells and transgenic mice. Nat. Med..

[cit35] Tomita T., Maruyama K., Saido T. C., Kume H., Shinozaki K., Tokuhiro S., Capell A., Walter J., Grünberg J., Haass C., Iwatsubo T., Obata K. (1997). The presenilin 2 mutation (N141I) linked to familial Alzheimer disease (Volga German families) increases the secretion of amyloid β protein ending at the 42nd (or 43rd) residue. Proc. Natl. Acad. Sci. U. S. A..

[cit36] Xia W., Zhang J., Kholodenko D., Citron M., Podlisny M. B., Teplow D. B., Haass C., Seubert P., Koo E. H., Selkoe D. J. (1997). Enhanced Production and Oligomerization of the 42-residue Amyloid β-Protein by Chinese Hamster Ovary Cells Stably Expressing Mutant Presenilins. J. Biol. Chem..

[cit37] Wolfe M. S., Xia W., Moore C. L., Leatherwood D. D., Ostaszewski B., Rahmati T., Donkor I. O., Selkoe D. J. (1999). Peptidomimetic Probes and Molecular Modeling Suggest That Alzheimer's γ-Secretase Is an Intramembrane-Cleaving Aspartyl Protease. Biochemistry.

[cit38] Wolfe M. S., Xia W., Ostaszewski B. L., Diehl T. S., Kimberly W. T., Selkoe D. J. (1999). Two transmembrane aspartates
in presenilin-1 required for presenilin endoproteolysis and γ-secretase activity. Nature.

[cit39] Kimberly W. T., Xia W., Rahmati T., Wolfe M. S., Selkoe D. J. (2000). The Transmembrane Aspartates in Presenilin 1 and 2 Are Obligatory for γ-Secretase Activity and Amyloid β-Protein Generation. J. Biol. Chem..

[cit40] Leimer U., Lun K., Romig H., Walter J., Grünberg J., Brand M., Haass C. (1999). Zebrafish (Danio rerio) presenilin promotes aberrant amyloid beta-peptide production and requires a critical aspartate residue for its function in amyloidogenesis. Biochemistry.

[cit41] Esler W. P., Kimberly W. T., Ostaszewski B. L., Diehl T. S., Moore C. L., Tsai J.-Y., Rahmati T., Xia W., Selkoe D. J., Wolfe M. S. (2000). Transition-state analogue inhibitors of γ-secretase bind directly to presenilin-1. Nat. Cell Biol..

[cit42] Li Y.-M., Xu M., Lai M.-T., Huang Q., Castro J. L., DiMuzio-Mower J., Harrison T., Lellis C., Nadin A., Neduvelil J. G., Register R. B., Sardana M. K., Shearman M. S., Smith A. L., Shi X.-P., Yin K.-C., Shafer J. A., Gardell S. J. (2000). Photoactivated γ-secretase inhibitors directed to the active site covalently label presenilin 1. Nature.

[cit43] De Strooper B., Annaert W., Cupers P., Saftig P., Craessaerts K., Mumm J. S., Schroeter E. H., Schrijvers V., Wolfe M. S., Ray W. J., Goate A., Kopan R. (1999). A presenilin-1-dependent γ-secretase-like protease mediates release of Notch intracellular domain. Nature.

[cit44] Schroeter E. H., Kisslinger J. A., Kopan R. (1998). Notch-1 signalling requires ligand-induced proteolytic release of intracellular domain. Nature.

[cit45] Struhl G., Greenwald I. (1999). Presenilin is required for activity and nuclear access of Notch in Drosophila. Nature.

[cit46] Song W., Nadeau P., Yuan M., Yang X., Shen J., Yankner B. A. (1999). Proteolytic release and nuclear translocation of Notch-1 are induced by presenilin-1 and impaired by pathogenic presenilin-1 mutations. Proc. Natl. Acad. Sci. U. S. A..

[cit47] Okochi M. (2002). Presenilins mediate a dual intramembranous gamma-secretase cleavage of Notch-1. EMBO J..

[cit48] Kimberly W. T., Esler W. P., Ye W., Ostaszewski B. L., Gao J., Diehl T., Selkoe D. J., Wolfe M. S. (2003). Notch and the Amyloid Precursor Protein Are Cleaved by Similar γ-Secretase(s. Biochemistry.

[cit49] Gu Y., Misonou H., Sato T., Dohmae N., Takio K., Ihara Y. (2001). Distinct Intramembrane Cleavage of the β-Amyloid Precursor Protein Family Resembling γ-Secretase-like Cleavage of Notch. J. Biol. Chem..

[cit50] Thinakaran G., Borchelt D. R., Lee M. K., Slunt H. H., Spitzer L., Kim G., Ratovitsky T., Davenport F., Nordstedt C., Seeger M., Hardy J., Levey A. I., Gandy S. E., Jenkins N. A., Copeland N. G., Price D. L., Sisodia S. S. (1996). Endoproteolysis of Presenilin 1 and Accumulation of Processed Derivatives *In Vivo*. Neuron.

[cit51] Ratovitski T., Slunt H. H., Thinakaran G., Price D. L., Sisodia S. S., Borchelt D. R. (1997). Endoproteolytic Processing and Stabilization of Wild-type and Mutant Presenilin. J. Biol. Chem..

[cit52] Podlisny M. B., Citron M., Amarante P., Sherrington R., Xia W., Zhang J., Diehl T., Levesque G., Fraser P., Haass C., Koo E. H. M., Seubert P., St. George-Hyslop P., Teplow D. B., Selkoe D. J. (1997). Presenilin Proteins Undergo Heterogeneous Endoproteolysis between Thr291and Ala299and Occur as Stable N- and C-Terminal Fragments in Normal and Alzheimer Brain Tissue. Neurobiol. Dis..

[cit53] Seeger M., Nordstedt C., Petanceska S., Kovacs D. M., Gouras G. K., Hahne S., Fraser P., Levesque L., Czernik A. J., George-Hyslop P. S., Sisodia S. S., Thinakaran G., Tanzi R. E., Greengard P., Gandy S. (1997). Evidence for phosphorylation and oligomeric assembly of presenilin 1. Proc. Natl. Acad. Sci. U. S. A..

[cit54] Capell A., Grünberg J., Pesold B., Diehlmann A., Citron M., Nixon R., Beyreuther K., Selkoe D. J., Haass C. (1998). The Proteolytic Fragments of the Alzheimer's Disease-associated Presenilin-1 Form Heterodimers and Occur as a 100–150-kDa Molecular Mass Complex. J. Biol. Chem..

[cit55] Yu G., Chen F., Levesque G., Nishimura M., Zhang D.-M., Levesque L., Rogaeva E., Xu D., Liang Y., Duthie M., George-Hyslop P. H. S., Fraser P. E. (1998). The Presenilin 1 Protein Is a Component of a High Molecular Weight Intracellular Complex That Contains β-Catenin. J. Biol. Chem..

[cit56] Yu G., Nishimura M., Arawaka S., Levitan D., Zhang L., Tandon A., Song Y.-Q., Rogaeva E., Chen F., Kawarai T., Supala A., Levesque L., Yu H., Yang D.-S., Holmes E., Milman P., Liang Y., Zhang D. M., Xu D. H. (2000). *et al.*, Nicastrin modulates presenilin-mediated notch/glp-1 signal transduction and βAPP processing. Nature.

[cit57] Li T., Ma G., Cai H., Price D. L., Wong P. C. (2003). Nicastrin Is Required for Assembly of Presenilin/γ-Secretase Complexes to Mediate Notch Signaling and for Processing and Trafficking of β-Amyloid Precursor Protein in Mammals. J. Neurosci..

[cit58] Lee S.-F., Shah S., Li H., Yu C., Han W., Yu G. (2002). Mammalian APH-1 Interacts with Presenilin and Nicastrin and Is Required for Intramembrane Proteolysis of Amyloid-β Precursor Protein and Notch. J. Biol. Chem..

[cit59] Goutte C., Tsunozaki M., Hale V. A., Priess J. R. (2002). APH-1 is a multipass membrane protein essential for the Notch signaling pathway in *Caenorhabditis elegans* embryos. Proc. Natl. Acad. Sci. U. S. A..

[cit60] Gu Y., Chen F., Sanjo N., Kawarai T., Hasegawa H., Duthie M., Li W., Ruan X., Luthra A., Mount H. T. J., Tandon A., Fraser P. E., St George-Hyslop P. (2003). APH-1 Interacts with Mature and Immature Forms of Presenilins and Nicastrin and May Play a Role in Maturation of Presenilin·Nicastrin Complexes. J. Biol. Chem..

[cit61] Francis R., McGrath G., Zhang J., Ruddy D. A., Sym M., Apfeld J., Nicoll M., Maxwell M., Hai B., Ellis M. C., Parks A. L., Xu W., Li J., Gurney M., Myers R. L., Himes C. S., Hiebsch R., Ruble C., Nye J. S., Curtis D. (2002). Aph-1 and pen-2 Are Required for Notch Pathway Signaling, γ-Secretase Cleavage of βAPP, and Presenilin Protein Accumulation. Dev. Cell.

[cit62] Steiner H., Winkler E., Edbauer D., Prokop S., Basset G., Yamasaki A., Kostka M., Haass C. (2002). PEN-2 Is an Integral Component of the γ-Secretase Complex Required for Coordinated Expression of Presenilin and Nicastrin. J. Biol. Chem..

[cit63] Kimberly W. T., LaVoie M. J., Ostaszewski B. L., Ye W., Wolfe M. S., Selkoe D. J. (2003). γ-Secretase is a membrane protein complex comprised of presenilin, nicastrin, aph-1, and pen-2. Proc. Natl. Acad. Sci. U. S. A..

[cit64] Edbauer D., Winkler E., Regula J. T., Pesold B., Steiner H., Haass C. (2003). Reconstitution of γ-secretase activity. Nat. Cell Biol..

[cit65] Takasugi N., Tomita T., Hayashi I., Tsuruoka M., Niimura M., Takahashi Y., Thinakaran G., Iwatsubo T. (2003). The role of presenilin cofactors in the γ-secretase complex. Nature.

[cit66] Sato T., Diehl T. S., Narayanan S., Funamoto S., Ihara Y., De Strooper B., Steiner H., Haass C., Wolfe M. S. (2007). Active γ-Secretase Complexes Contain Only One of Each Component. J. Biol. Chem..

[cit67] Gertsik N., Chiu D., Li Y.-M. (2015). Complex regulation of Î^3^-secretase: From obligatory to modulatory subunits. Front. Aging Neurosci..

[cit68] Haapasalo A., Kovacs D. M. (2011). The Many Substrates of Presenilin/γ-Secretase. J. Alzheimer's Dis..

[cit69] Laudon H., Hansson E. M., Melén K., Bergman A., Farmery M. R., Winblad B., Lendahl U., Von Heijne G., Näslund J. (2005). A Nine-transmembrane Domain Topology for Presenilin 1. J. Biol. Chem..

[cit70] Wang J., Brunkan A. L., Hecimovic S., Walker E., Goate A. (2004). Conserved “PAL” sequence in presenilins is essential for γ-secretase activity, but not required for formation or stabilization of γ-secretase complexes. Neurobiol. Dis..

[cit71] Wang J., Beher D., Nyborg A. C., Shearman M. S., Golde T. E., Goate A. (2006). C-terminal PAL motif of presenilin and presenilin homologues required for normal active site conformation. J. Neurochem..

[cit72] Sato C., Takagi S., Tomita T., Iwatsubo T. (2008). The C-Terminal PAL Motif and Transmembrane Domain 9 of Presenilin 1 Are Involved in the Formation of the Catalytic Pore of the -Secretase. J. Neurosci..

[cit73] Yamasaki A., Eimer S., Okochi M., Smialowska A., Kaether C., Baumeister R., Haass C., Steiner H. (2006). The GxGD Motif of Presenilin Contributes to Catalytic Function and Substrate Identification of γ-Secretase. J. Neurosci..

[cit74] Kretner B., Fukumori A., Kuhn P., Pérez-Revuelta B. I., Lichtenthaler S. F., Haass C., Steiner H. (2013). Important functional role of residue x of the presenilin Gx GD protease active site motif for APP substrate cleavage specificity and substrate selectivity of γ-secretase. J. Neurochem..

[cit75] Carroll C. M., Li Y.-M. (2016). Physiological and pathological roles of the γ-secretase complex. Brain Res. Bull..

[cit76] Shah S., Lee S.-F., Tabuchi K., Hao Y.-H., Yu C., LaPlant Q., Ball H., Dann C. E., Südhof T., Yu G. (2005). Nicastrin Functions as a γ-Secretase-Substrate Receptor. Cell.

[cit77] Bolduc D. M., Montagna D. R., Gu Y., Selkoe D. J., Wolfe M. S. (2016). Nicastrin functions to sterically hinder γ-secretase–substrate interactions driven by substrate transmembrane domain. Proc. Natl. Acad. Sci. U. S. A..

[cit78] Zhao G., Liu Z., Ilagan Ma. X. G., Kopan R. (2010). γ-Secretase Composed of PS1/Pen2/Aph1a Can Cleave Notch and Amyloid Precursor Protein in the Absence of Nicastrin. J. Neurosci..

[cit79] Hu C., Zeng L., Li T., Meyer M. A., Cui M., Xu X. (2016). Nicastrin is required for amyloid precursor protein (APP) but not Notch processing, while anterior pharynx-defective 1 is dispensable for processing of both APP and Notch. J. Neurochem..

[cit80] Niimura M., Isoo N., Takasugi N., Tsuruoka M., Ui-Tei K., Saigo K., Morohashi Y., Tomita T., Iwatsubo T. (2005). Aph-1 Contributes to the Stabilization and Trafficking of the γ-Secretase Complex through Mechanisms Involving Intermolecular and Intramolecular Interactions. J. Biol. Chem..

[cit81] Fortna R. R., Crystal A. S., Morais V. A., Pijak D. S., Lee V. M.-Y., Doms R. W. (2004). Membrane Topology and Nicastrin-enhanced Endoproteolysis of APH-1, a Component of the γ-Secretase Complex. J. Biol. Chem..

[cit82] Crystal A. S., Morais V. A., Pierson T. C., Pijak D. S., Carlin D., Lee V. M.-Y., Doms R. W. (2003). Membrane Topology of γ-Secretase Component PEN-2. J. Biol. Chem..

[cit83] Prokop S., Shirotani K., Edbauer D., Haass C., Steiner H. (2004). Requirement of PEN-2 for Stabilization of the Presenilin N-/C-terminal Fragment Heterodimer within the γ-Secretase Complex. J. Biol. Chem..

[cit84] Holmes O., Paturi S., Selkoe D. J., Wolfe M. S. (2014). Pen-2 Is Essential for γ-Secretase Complex Stability and Trafficking but Partially Dispensable for Endoproteolysis. Biochemistry.

[cit85] Watanabe N., Tomita T., Sato C., Kitamura T., Morohashi Y., Iwatsubo T. (2005). Pen-2 Is Incorporated into the γ-Secretase Complex through Binding to Transmembrane Domain 4 of Presenilin 1. J. Biol. Chem..

[cit86] Kim S.-H., Sisodia S. S. (2005). A Sequence within the First Transmembrane Domain of PEN-2 Is Critical for PEN-2-mediated Endoproteolysis of Presenilin 1. J. Biol. Chem..

[cit87] Wolfe M. S. (2019). Structure and Function of the γ-Secretase Complex. Biochemistry.

[cit88] Lu P., Bai X., Ma D., Xie T., Yan C., Sun L., Yang G., Zhao Y., Zhou R., Scheres S. H. W., Shi Y. (2014). Three-dimensional structure of human γ-secretase. Nature.

[cit89] Bai X., Yan C., Yang G., Lu P., Ma D., Sun L., Zhou R., Scheres S. H. W., Shi Y. (2015). An atomic structure of human γ-secretase. Nature.

[cit90] Wolfe M. S. (2020). Substrate recognition and processing by γ-secretase. Biochim. Biophys. Acta, Biomembr..

[cit91] Sun L., Zhao L., Yang G., Yan C., Zhou R., Zhou X., Xie T., Zhao Y., Wu S., Li X., Shi Y. (2015). Structural basis of human γ-secretase assembly. Proc. Natl. Acad. Sci. U. S. A..

[cit92] Yang G., Zhou R., Shi Y. (2017). Cryo-EM structures of human γ-secretase. Curr. Opin. Struct. Biol..

[cit93] Zhou R., Yang G., Guo X., Zhou Q., Lei J., Shi Y. (2019). Recognition of the amyloid precursor protein by human γ-secretase. Science.

[cit94] Yang G., Zhou R., Zhou Q., Guo X., Yan C., Ke M., Lei J., Shi Y. (2019). Structural basis of Notch recognition by human γ-secretase. Nature.

[cit95] Bai X., Rajendra E., Yang G., Shi Y., Scheres S. H. (2015). Sampling the conformational space of the catalytic subunit of human γ-secretase. eLife.

[cit96] Yang G., Zhou R., Guo X., Yan C., Lei J., Shi Y. (2021). Structural basis of γ-secretase inhibition and modulation by small molecule drugs. Cell.

[cit97] Hitzenberger M., Götz A., Menig S., Brunschweiger B., Zacharias M., Scharnagl C. (2020). The dynamics of γ-secretase and its substrates. Semin. Cell Dev. Biol..

[cit98] Miao Y., Wolfe M. S. (2025). Emerging structures and dynamic mechanisms of γ-secretase for Alzheimer's disease. Neural Regen. Res..

[cit99] Bhattarai A., Devkota S., Bhattarai S., Wolfe M. S., Miao Y. (2020). Mechanisms of γ-Secretase Activation and Substrate Processing. ACS Cent. Sci..

[cit100] Do H. N., Malvankar S. R., Wolfe M. S., Miao Y. (2023). Molecular Dynamics Activation of γ-Secretase for Cleavage of the Notch1 Substrate. ACS Chem. Neurosci..

[cit101] Güner G., Lichtenthaler S. F. (2020). The substrate repertoire of γ-secretase/presenilin. Semin. Cell Dev. Biol..

[cit102] Hemming M. L., Elias J. E., Gygi S. P., Selkoe D. J. (2008). Proteomic Profiling of γ-Secretase Substrates and Mapping of Substrate Requirements. PLoS Biol..

[cit103] Struhl G., Adachi A. (2000). Requirements for Presenilin-Dependent Cleavage of Notch and Other Transmembrane Proteins. Mol. Cell.

[cit104] Beel A. J., Sanders C. R. (2008). Substrate specificity of γ-secretase and other intramembrane proteases. Cell. Mol. Life Sci..

[cit105] Qi-Takahara Y., Morishima-Kawashima M., Tanimura Y., Dolios G., Hirotani N., Horikoshi Y., Kametani F., Maeda M., Saido T. C., Wang R., Ihara Y. (2005). Longer Forms of Amyloid β Protein: Implications for the Mechanism of Intramembrane Cleavage by γ-Secretase. J. Neurosci..

[cit106] Lammich S., Okochi M., Takeda M., Kaether C., Capell A., Zimmer A.-K., Edbauer D., Walter J., Steiner H., Haass C. (2002). Presenilin-dependent Intramembrane Proteolysis of CD44 Leads to the Liberation of Its Intracellular Domain and the Secretion of an Aβ-like Peptide. J. Biol. Chem..

[cit107] Kopan R., Ilagan Ma. X. G. (2004). γ-Secretase: Proteasome of the membrane. Nat. Rev. Mol. Cell Biol..

[cit108] Wolfe M. S. (2009). γ-Secretase in biology and medicine. Semin. Cell Dev. Biol..

[cit109] Zheng H., Koo E. H. (2011). Biology and pathophysiology of the amyloid precursor protein. Mol. Neurodegener..

[cit110] Deyts C., Thinakaran G., Parent A. T. (2016). APP Receptor? To Be or Not To Be. Trends Pharmacol. Sci..

[cit111] Wolfe M. S., Guénette S. Y. (2007). APP at a glance. J. Cell Sci..

[cit112] Vassar R., Bennett B. D., Babu-Khan S., Kahn S., Mendiaz E. A., Denis P., Teplow D. B., Ross S., Amarante P., Loeloff R., Luo Y., Fisher S., Fuller J., Edenson S., Lile J., Jarosinski M. A., Biere A. L., Curran E., Burgess T. (1999). *et al.*, β-Secretase Cleavage of Alzheimer's Amyloid Precursor Protein by the Transmembrane Aspartic Protease BACE. Science.

[cit113] Hussain I., Powell D., Howlett D. R., Tew D. G., Meek T. D., Chapman C., Gloger I. S., Murphy K. E., Southan C. D., Ryan D. M., Smith T. S., Simmons D. L., Walsh F. S., Dingwall C., Christie G. (1999). Identification of a Novel Aspartic Protease (Asp 2) as β-Secretase. Mol. Cell. Neurosci..

[cit114] Yan R., Bienkowski M. J., Shuck M. E., Miao H., Tory M. C., Pauley A. M., Brashler J. R., Stratman N. C., Mathews W. R., Buhl A. E., Carter D. B., Tomasselli A. G., Parodi L. A., Heinrikson R. L., Gurney M. E. (1999). Membrane-anchored aspartyl protease with Alzheimer's disease β-secretase activity. Nature.

[cit115] Sinha S., Anderson J. P., Barbour R., Basi G. S., Caccavello R., Davis D., Doan M., Dovey H. F., Frigon N., Hong J., Jacobson-Croak K., Jewett N., Keim P., Knops J., Lieberburg I., Power M., Tan H., Tatsuno G., Tung J. (1999). *et al.*, Purification and cloning of amyloid precursor protein β-secretase from human brain. Nature.

[cit116] Lin X., Koelsch G., Wu S., Downs D., Dashti A., Tang J. (2000). Human aspartic protease memapsin 2 cleaves the β-secretase site of β-amyloid precursor protein. Proc. Natl. Acad. Sci. U. S. A..

[cit117] Thinakaran G., Koo E. H. (2008). Amyloid Precursor Protein Trafficking, Processing, and Function. J. Biol. Chem..

[cit118] Checler F., Afram E., Pardossi-Piquard R., Lauritzen I. (2021). Is γ-secretase a beneficial inactivating enzyme of the toxic APP C-terminal fragment C99. J. Biol. Chem..

[cit119] Castro M. A., Hadziselimovic A., Sanders C. R. (2019). The vexing complexity of the amyloidogenic pathway. Protein Sci..

[cit120] Ling Y., Morgan K., Kalsheker N. (2003). Amyloid precursor protein (APP) and the biology of proteolytic processing: Relevance to Alzheimer's disease. Int. J. Biochem. Cell Biol..

[cit121] Bray S. J. (2006). Notch signalling: A simple pathway becomes complex. Nat. Rev. Mol. Cell Biol..

[cit122] Siebel C., Lendahl U. (2017). Notch Signaling in Development, Tissue Homeostasis, and Disease. Physiol. Rev..

[cit123] Meng Y., Bo Z., Feng X., Yang X., Handford P. A. (2024). The Notch Signaling Pathway: Mechanistic Insights in Health and Disease. Engineering.

[cit124] Shi Q., Xue C., Zeng Y., Yuan X., Chu Q., Jiang S., Wang J., Zhang Y., Zhu D., Li L. (2024). Notch signaling pathway in cancer: From mechanistic insights to targeted therapies. Signal Transduct. Target. Ther..

[cit125] Brou C., Logeat F., Gupta N., Bessia C., LeBail O., Doedens J. R., Cumano A., Roux P., Black R. A., Israël A. (2000). A Novel Proteolytic Cleavage Involved in Notch Signaling. Mol. Cell.

[cit126] Henrique D., Schweisguth F. (2019). Mechanisms of Notch signaling: A simple logic deployed in time and space. Development.

[cit127] Mumm J. S., Schroeter E. H., Saxena M. T., Griesemer A., Tian X., Pan D. J., Ray W. J., Kopan R. (2000). A Ligand-Induced Extracellular Cleavage Regulates γ-Secretase-like Proteolytic Activation of Notch1. Mol. Cell.

[cit128] Malvankar S. R., Wolfe M. S. (2025). Effects of Transmembrane Phenylalanine Residues on γ-Secretase-Mediated Notch-1 Proteolysis. ACS Chem. Neurosci..

[cit129] Struhl G., Adachi A. (1998). Nuclear Access and Action of Notch *In Vivo*. Cell.

[cit130] Laurent S. A., Hoffmann F. S., Kuhn P.-H., Cheng Q., Chu Y., Schmidt-Supprian M., Hauck S. M., Schuh E., Krumbholz M., Rübsamen H., Wanngren J., Khademi M., Olsson T., Alexander T., Hiepe F., Pfister H.-W., Weber F., Jenne D., Wekerle H. (2015). *et al.*, γ-secretase directly sheds the survival receptor BCMA from plasma cells. Nat. Commun..

[cit131] Glebov K., Wunderlich P., Karaca I., Walter J. (2016). Functional involvement of γ-secretase in signaling of the triggering receptor expressed on myeloid cells-2 (TREM2. J. Neuroinflammation.

[cit132] Zhong L., Chen X.-F., Wang T., Wang Z., Liao C., Wang Z., Huang R., Wang D., Li X., Wu L., Jia L., Zheng H., Painter M., Atagi Y., Liu C.-C., Zhang Y.-W., Fryer J. D., Xu H., Bu G. (2017). Soluble TREM2 induces inflammatory responses and enhances microglial survival. J. Exp. Med..

[cit133] Chhibber-Goel J., Coleman-Vaughan C., Agrawal V., Sawhney N., Hickey E., Powell J. C., McCarthy J. V. (2016). γ-Secretase Activity Is Required for Regulated Intramembrane Proteolysis of Tumor Necrosis Factor (TNF) Receptor 1 and TNF-mediated Pro-apoptotic Signaling. J. Biol. Chem..

[cit134] Murakami D., Okamoto I., Nagano O., Kawano Y., Tomita T., Iwatsubo T., De Strooper B., Yumoto E., Saya H. (2003). Presenilin-dependent γ-secretase activity mediates the intramembranous cleavage of CD44. Oncogene.

[cit135] Ponta H., Sherman L., Herrlich P. A. (2003). CD44: From adhesion molecules to signalling regulators. Nat. Rev. Mol. Cell Biol..

[cit136] Marambaud P. (2002). A presenilin-1/gamma-secretase cleavage releases the E-cadherin intracellular domain and regulates disassembly of adherens junctions. EMBO J..

[cit137] Marambaud P., Wen P. H., Dutt A., Shioi J., Takashima A., Siman R., Robakis N. K. (2003). A CBP Binding Transcriptional Repressor Produced by the PS1/ε-Cleavage of N-Cadherin Is Inhibited by PS1 FAD Mutations. Cell.

[cit138] Shapiro L., Weis W. I. (2009). Structure and Biochemistry of Cadherins and Catenins. Cold Spring Harbor Perspect. Biol..

[cit139] Oda H., Takeichi M. (2011). Structural and functional diversity of cadherin at the adherens junction. J. Cell Biol..

[cit140] Craig A. M., Kang Y. (2007). Neurexin–neuroligin signaling in synapse development. Curr. Opin. Neurobiol..

[cit141] Südhof T. C. (2008). Neuroligins and neurexins link synaptic function to cognitive disease. Nature.

[cit142] Saura C. A., Servián-Morilla E., Scholl F. G. (2011). Presenilin/γ-Secretase Regulates Neurexin Processing at Synapses. PLoS One.

[cit143] Bot N., Schweizer C., Ben Halima S., Fraering P. C. (2011). Processing of the Synaptic Cell Adhesion Molecule Neurexin-3β by Alzheimer Disease α- and γ-Secretases. J. Biol. Chem..

[cit144] Suzuki K., Hayashi Y., Nakahara S., Kumazaki H., Prox J., Horiuchi K., Zeng M., Tanimura S., Nishiyama Y., Osawa S., Sehara-Fujisawa A., Saftig P., Yokoshima S., Fukuyama T., Matsuki N., Koyama R., Tomita T., Iwatsubo T. (2012). Activity-Dependent Proteolytic Cleavage of Neuroligin-1. Neuron.

[cit145] Peixoto R. T., Kunz P. A., Kwon H., Mabb A. M., Sabatini B. L., Philpot B. D., Ehlers M. D. (2012). Transsynaptic Signaling by Activity-Dependent Cleavage of Neuroligin-1. Neuron.

[cit146] Longart M., Calderón C., González M., Grela M. E., Martínez J. C. (2022). Neuregulins: Subcellular localization, signaling pathways and their relationship with neuroplasticity and neurological diseases. Explor. Neurosci..

[cit147] Merilahti J. A. M., Ojala V. K., Knittle A. M., Pulliainen A. T., Elenius K. (2017). Genome-wide screen of gamma-secretase–mediated intramembrane cleavage of receptor tyrosine kinases. Mol. Biol. Cell.

[cit148] Wolfe M. S. (2022). γ-Secretase As a Drug Target for Familial Alzheimer's Disease: The Road Less Traveled. Future Med. Chem..

[cit149] Gu Y., Misonou H., Sato T., Dohmae N., Takio K., Ihara Y. (2001). Distinct Intramembrane Cleavage of the β-Amyloid Precursor Protein Family Resembling γ-Secretase-like Cleavage of Notch. J. Biol. Chem..

[cit150] Weidemann A., Eggert S., Reinhard F. B. M., Vogel M., Paliga K., Baier G., Masters C. L., Beyreuther K., Evin G. (2002). A Novel ε-Cleavage within the Transmembrane Domain of the Alzheimer Amyloid Precursor Protein Demonstrates Homology with Notch Processing. Biochemistry.

[cit151] Sato T., Dohmae N., Qi Y., Kakuda N., Misonou H., Mitsumori R., Maruyama H., Koo E. H., Haass C., Takio K., Morishima-Kawashima M., Ishiura S., Ihara Y. (2003). Potential Link between Amyloid β-Protein 42 and C-terminal Fragment γ 49–99 of β-Amyloid Precursor Protein. J. Biol. Chem..

[cit152] Kakuda N., Funamoto S., Yagishita S., Takami M., Osawa S., Dohmae N., Ihara Y. (2006). Equimolar Production of Amyloid β-Protein and Amyloid Precursor Protein Intracellular Domain from β-Carboxyl-terminal Fragment by γ-Secretase. J. Biol. Chem..

[cit153] Funamoto S., Morishima-Kawashima M., Tanimura Y., Hirotani N., Saido T. C., Ihara Y. (2004). Truncated Carboxyl-Terminal Fragments of β-Amyloid Precursor Protein Are Processed to Amyloid β-Proteins 40 and 42. Biochemistry.

[cit154] Yagishita S., Morishima-Kawashima M., Ishiura S., Ihara Y. (2008). Aβ46 Is Processed to Aβ40 and Aβ43, but
Not to Aβ42, in the Low Density Membrane Domains. J. Biol. Chem..

[cit155] Takami M., Nagashima Y., Sano Y., Ishihara S., Morishima-Kawashima M., Funamoto S., Ihara Y. (2009). γ-Secretase: Successive Tripeptide and Tetrapeptide Release from the Transmembrane Domain of β-Carboxyl Terminal Fragment. J. Neurosci..

[cit156] Okochi M., Tagami S., Yanagida K., Takami M., Kodama T. S., Mori K., Nakayama T., Ihara Y., Takeda M. (2013). γ-Secretase Modulators and Presenilin 1 Mutants Act Differently on Presenilin/γ-Secretase Function to Cleave Aβ42 and Aβ43. Cell Rep..

[cit157] Matsumura N., Takami M., Okochi M., Wada-Kakuda S., Fujiwara H., Tagami S., Funamoto S., Ihara Y., Morishima-Kawashima M. (2014). γ-Secretase Associated with Lipid Rafts. J. Biol. Chem..

[cit158] Moore C. L., Leatherwood D. D., Diehl T. S., Selkoe D. J., Wolfe M. S. (2000). Difluoro Ketone Peptidomimetics Suggest a Large S1 Pocket for Alzheimer's γ-Secretase: Implications for Inhibitor Design. J. Med. Chem..

[cit159] Esler W. P., Das C., Wolfe M. S. (2004). Probing pockets S2–S4′ of the γ-secretase active site with (hydroxyethyl)urea peptidomimetics. Bioorg. Med. Chem. Lett..

[cit160] Bolduc D. M., Montagna D. R., Seghers M. C., Wolfe M. S., Selkoe D. J. (2016). The amyloid-beta forming tripeptide cleavage mechanism of γ-secretase. eLife.

[cit161] Guo X., Li H., Yan C., Lei J., Zhou R., Shi Y. (2024). Molecular mechanism of substrate recognition and cleavage by human γ-secretase. Science.

[cit162] Malvankar S. R., Wolfe M. S. (2025). γ-Secretase-Mediated Endoproteolysis of Neuregulin-1 and E-Cadherin. Biochemistry.

[cit163] Scheltens P., Strooper B. D., Kivipelto M., Holstege H., Chételat G., Teunissen C. E., Cummings J., van der Flier W. M. (2021). Alzheimer's disease. Lancet.

[cit164] Liyanage S. I. and Weaver D. F., Misfolded proteins as a therapeutic target in Alzheimer's disease, in Advances in Protein Chemistry and Structural Biology, Elsevier, 2019, vol. 118, pp. 371–411 10.1016/bs.apcsb.2019.08.003 31928732

[cit165] Quan M., Cao S., Wang Q., Wang S., Jia J. (2023). Genetic Phenotypes of Alzheimer's Disease: Mechanisms and Potential Therapy. Phenomics.

[cit166] Tang Y., Zhang D., Gong X., Zheng J. (2022). A mechanistic survey of Alzheimer's disease. Biophys. Chem..

[cit167] Liu P.-P., Xie Y., Meng X.-Y., Kang J.-S. (2019). History and progress of hypotheses and clinical trials for Alzheimer's disease. Signal Transduct. Target. Ther..

[cit168] Zhang J., Zhang Y., Wang J., Xia Y., Zhang J., Chen L. (2024). Recent advances in Alzheimer's disease: Mechanisms, clinical trials and new drug development strategies. Signal Transduct. Target. Ther..

[cit169] Madav Y., Wairkar S., Prabhakar B. (2019). Recent therapeutic strategies targeting beta amyloid and tauopathies in Alzheimer's disease. Brain Res. Bull..

[cit170] Gupta G. L., Samant N. P. (2021). Current druggable targets for therapeutic control of Alzheimer's disease. Contemp. Clin. Trials.

[cit171] Cummings J. L., Zhou Y., Yang Y., Zhong K., Fonseca J., Osse A. L., Cheng F. (2026). Alzheimer's disease drug development pipeline: 2026. Alzheimers Dement.: Transl. Res. Clin. Interventions.

[cit172] Hardy J., Allsop D. (1991). Amyloid deposition as the central event in the aetiology of Alzheimer's disease. Trends Pharmacol. Sci..

[cit173] Hardy J. A., Higgins G. A. (1992). Alzheimer's Disease: The Amyloid Cascade Hypothesis. Science.

[cit174] Selkoe D. J. (1991). The molecular pathology of Alzheimer's disease. Neuron.

[cit175] Selkoe D. J., Hardy J. (2016). The amyloid hypothesis of Alzheimer's disease at 25 years. EMBO Mol. Med..

[cit176] Hardy J. (2009). The amyloid hypothesis for Alzheimer's disease: A critical reappraisal. J. Neurochem..

[cit177] Iwatsubo T., Odaka A., Suzuki N., Mizusawa H., Nukina N., Ihara Y. (1994). Visualization of Aβ42(43) and Aβ40 in senile plaques with end-specific Aβ monoclonals: Evidence that an initially deposited species is Aβ42(43. Neuron.

[cit178] Roher A. E., Lowenson J. D., Clarke S., Woods A. S., Cotter R. J., Gowing E., Ball M. J. (1993). beta-Amyloid-(1-42) is a major component of cerebrovascular amyloid deposits: Implications for the pathology of Alzheimer disease. Proc. Natl. Acad. Sci. U. S. A..

[cit179] Scheuner D., Eckman C., Jensen M., Song X., Citron M., Suzuki N., Bird T. D., Hardy J., Hutton M., Kukull W., Larson E., Levy-Lahad L., Viitanen M., Peskind E., Poorkaj P., Schellenberg G., Tanzi R., Wasco W., Lannfelt L. (1996). *et al.*, Secreted amyloid β–protein similar to that in the senile plaques of Alzheimer's disease is increased *in vivo* by the presenilin 1 and 2 and APP mutations linked to familial Alzheimer's disease. Nat. Med..

[cit180] Selkoe D. J. (2001). Alzheimer's Disease: Genes, Proteins, and Therapy. Physiol. Rev..

[cit181] Younkin S. G. (1998). The role of Aβ42 in Alzheimer's disease. J. Physiol..

[cit182] Potter R., Patterson B. W., Elbert D. L., Ovod V., Kasten T., Sigurdson W., Mawuenyega K., Blazey T., Goate A., Chott R., Yarasheski K. E., Holtzman D. M., Morris J. C., Benzinger T. L. S., Bateman R. J. (2013). Increased *in Vivo* Amyloid-β42 Production, Exchange, and Loss in Presenilin Mutation Carriers. Sci. Transl. Med..

[cit183] Mawuenyega K. G., Sigurdson W., Ovod V., Munsell L., Kasten T., Morris J. C., Yarasheski K. E., Bateman R. J. (2010). Decreased Clearance of CNS β-Amyloid in Alzheimer's Disease. Science.

[cit184] Patterson B. W., Elbert D. L., Mawuenyega K. G., Kasten T., Ovod V., Ma S., Xiong C., Chott R., Yarasheski K., Sigurdson W., Zhang L., Goate A., Benzinger T., Morris J. C., Holtzman D., Bateman R. J. (2015). Age and amyloid effects on human central nervous system amyloid-beta kinetics. Ann. Neurol..

[cit185] Lane C. A., Hardy J., Schott J. M. (2018). Alzheimer's disease. Eur. J. Neurol..

[cit186] Jonsson T., Atwal J. K., Steinberg S., Snaedal J., Jonsson P. V., Bjornsson S., Stefansson H., Sulem P., Gudbjartsson D., Maloney J., Hoyte K., Gustafson A., Liu Y., Lu Y., Bhangale T., Graham R. R., Huttenlocher J., Bjornsdottir G., Andreassen O. A. (2012). *et al.*, A mutation in APP protects against Alzheimer's disease and age-related cognitive decline. Nature.

[cit187] Watkins E. A., Vassar R. (2024). BACE Inhibitor Clinical Trials for Alzheimer's Disease. J. Alzheimer's Dis..

[cit188] Hur J.-Y. (2022). γ-Secretase in Alzheimer's disease. Exp. Mol. Med..

[cit189] Bateman R. J., Xiong C., Benzinger T. L. S., Fagan A. M., Goate A., Fox N. C., Marcus D. S., Cairns N. J., Xie X., Blazey T. M., Holtzman D. M., Santacruz A., Buckles V., Oliver A., Moulder K., Aisen P. S., Ghetti B., Klunk W. E., McDade E. (2012). *et al.*, Clinical and Biomarker Changes in Dominantly Inherited Alzheimer's Disease. N. Engl. J. Med..

[cit190] Yadollahikhales G., Rojas J. C. (2023). Anti-Amyloid Immunotherapies for Alzheimer's Disease: A 2023 Clinical Update. Neurotherapeutics.

[cit191] Cummings J. L. (2025). Maximizing the benefit and managing the risk of anti-amyloid monoclonal antibody therapy for Alzheimer's disease: Strategies and research directions. Neurotherapeutics.

[cit192] Cummings J. (2023). Anti-Amyloid Monoclonal Antibodies are Transformative Treatments that Redefine Alzheimer's Disease Therapeutics. Drugs.

[cit193] Cummings J., Osse A. M. L., Cammann D., Powell J., Chen J. (2024). Anti-Amyloid Monoclonal Antibodies for the Treatment of Alzheimer's Disease. BioDrugs.

[cit194] Mullard A. (2023). FDA approves second anti-amyloid antibody for Alzheimer disease. Nat. Rev. Drug Discovery.

[cit195] Reardon S. (2024). Alzheimer's drug with modest benefits wins backing of FDA advisers. Nature.

[cit196] Rafii M. S., Aisen P. S. (2025). Amyloid-lowering immunotherapies for Alzheimer disease: Current status and future directions. Nat. Rev. Neurol..

[cit197] Llibre-Guerra J. J., McDade E. M., Schindler S. E., Clifford D. B., Supnet C., Atri A., Bateman R. J. (2025). Towards pharmacological prevention of Alzheimer disease. Nat. Rev. Neurol..

[cit198] Bateman R. J., Li Y., McDade E. M., Llibre-Guerra J. J., Clifford D. B., Atri A., Mills S. L., Santacruz A. M., Wang G., Supnet C., Benzinger T. L. S., Gordon B. A., Ibanez L., Klein G., Baudler M., Doody R. S., Delmar P., Kerchner G. A., Bittner T. (2025). *et al.*, Safety and efficacy of long-term gantenerumab treatment in dominantly inherited Alzheimer's disease: An open-label extension of the phase 2/3 multicentre, randomised, double-blind, placebo-controlled platform DIAN-TU trial. Lancet Neurol..

[cit199] Devkota S., Williams T. D., Wolfe M. S. (2021). Familial Alzheimer's disease mutations in amyloid protein precursor alter proteolysis by γ-secretase to increase amyloid β-peptides of ≥45 residues. J. Biol. Chem..

[cit200] Sun L., Zhou R., Yang G., Shi Y. (2017). Analysis of 138 pathogenic mutations in presenilin-1 on the *in vitro* production of Aβ42 and Aβ40 peptides by γ-secretase. Proc. Natl. Acad. Sci. U. S. A..

[cit201] Castellani R. J., Plascencia-Villa G., Perry G. (2019). The amyloid cascade and Alzheimer's disease therapeutics: Theory *versus* observation. Lab. Invest..

[cit202] Ye J., Wan H., Chen S., Liu G.-P. (2024). Targeting tau in Alzheimer's disease: From mechanisms to clinical therapy. Neural Regen. Res..

[cit203] Chu D., Liu F. (2019). Pathological Changes of Tau Related to Alzheimer's Disease. ACS Chem. Neurosci..

[cit204] Zhang X., Wang J., Zhang Z., Ye K. (2024). Tau in neurodegenerative diseases: Molecular mechanisms, biomarkers, and therapeutic strategies. Transl. Neurodegener..

[cit205] Gu J., Liu F. (2020). Tau in Alzheimer's Disease: Pathological Alterations and an Attractive Therapeutic Target. Curr. Med. Sci..

[cit206] Reiss A. B., Gulkarov S., Jacob B., Srivastava A., Pinkhasov A., Gomolin I. H., Stecker M. M., Wisniewski T., De Leon J. (2024). Mitochondria in Alzheimer's Disease Pathogenesis. Life.

[cit207] Swerdlow R. H., Khan S. M. (2004). A “mitochondrial cascade hypothesis” for sporadic Alzheimer's disease. Med. Hypotheses.

[cit208] Swerdlow R. H., Burns J. M., Khan S. M. (2014). The Alzheimer's disease mitochondrial cascade hypothesis: Progress and perspectives. Biochim. Biophys. Acta, Mol. Basis Dis..

[cit209] Tönnies E., Trushina E. (2017). Oxidative Stress, Synaptic Dysfunction, and Alzheimer's Disease. J. Alzheimer's Dis..

[cit210] Singh A., Kukreti R., Saso L., Kukreti S. (2019). Oxidative Stress: A Key Modulator in Neurodegenerative Diseases. Molecules.

[cit211] Chen X., Yan S. D. (2006). Mitochondrial Aβ A potential cause of metabolic dysfunction in Alzheimer's disease. IUBMB Life.

[cit212] Coskun P. E., Beal M. F., Wallace D. C. (2004). Alzheimer's brains harbor somatic mtDNA control-region mutations that suppress mitochondrial transcription and replication. Proc. Natl. Acad. Sci. U. S. A..

[cit213] Kim J., Basak J. M., Holtzman D. M. (2009). The Role of Apolipoprotein E in Alzheimer's Disease. Neuron.

[cit214] Wisniewski T., Drummond E. (2020). APOE-amyloid interaction: Therapeutic targets. Neurobiol. Dis..

[cit215] Liu C.-C., Kanekiyo T., Xu H., Bu G. (2013). Apolipoprotein E and Alzheimer disease: Risk, mechanisms and therapy. Nat. Rev. Neurol..

[cit216] Brecht W. J., Harris F. M., Chang S., Tesseur I., Yu G.-Q., Xu Q., Dee Fish J., Wyss-Coray T., Buttini M., Mucke L., Mahley R. W., Huang Y. (2004). Neuron-Specific Apolipoprotein E4 Proteolysis Is Associated with Increased Tau Phosphorylation in Brains of Transgenic Mice. J. Neurosci..

[cit217] Ulrich J. D., Ulland T. K., Colonna M., Holtzman D. M. (2017). Elucidating the Role of TREM2 in Alzheimer's Disease. Neuron.

[cit218] Devkota S., Zhou R., Nagarajan V., Maesako M., Do H., Noorani A., Overmeyer C., Bhattarai S., Douglas J. T., Saraf A., Miao Y., Ackley B. D., Shi Y., Wolfe M. S. (2024). Familial Alzheimer mutations stabilize synaptotoxic γ-secretase-substrate complexes. Cell Rep..

[cit219] Wolfe M. S. (2025). Presenilin, γ-Secretase, and the Search for Pathogenic Triggers of Alzheimer's Disease. Biochemistry.

[cit220] Wang B., Yang W., Wen W., Sun J., Su B., Liu B., Ma D., Lv D., Wen Y., Qu T., Chen M., Sun M., Shen Y., Zhang X. (2010). γ-Secretase Gene Mutations in Familial Acne Inversa. Science.

[cit221] Nagarajan V. , Libowitz C. L., Ackley B. D. and Wolfe M. S., A C. elegans model of familial Alzheimer's disease shows age-dependent synaptic degeneration independent of amyloid β-peptide, 2025 10.1101/2025.07.16.665161 PMC13151068 41525885

[cit222] Yan K., Zhang C., Kang J., Montenegro P., Shen J. (2024). Cortical neurodegeneration caused by *Psen1* mutations is independent of Aβ. Proc. Natl. Acad. Sci. U. S. A..

[cit223] Rius-Pérez S., Tormos A. M., Pérez S., Taléns-Visconti R. (2018). Vascular pathology: Cause or effect in Alzheimer disease?. Neurologia (Engl. Ed.).

[cit224] Solis E., Hascup K. N., Hascup E. R. (2020). Alzheimer's Disease: The Link Between Amyloid-β and Neurovascular Dysfunction. J. Alzheimer's Dis..

[cit225] Wierenga C. E., Hays C. C., Zlatar Z. Z. (2014). Cerebral Blood Flow Measured by Arterial Spin Labeling MRI as a Preclinical Marker of Alzheimer's Disease. J. Alzheimer's Dis..

[cit226] Scheffer S., Hermkens D. M. A., Van Der Weerd L., De Vries H. E., Daemen M. J. A. P. (2021). Vascular Hypothesis of Alzheimer Disease: Topical Review of Mouse Models. Arterioscler., Thromb., Vasc. Biol..

[cit227] Fulop T., Witkowski J. M., Bourgade K., Khalil A., Zerif E., Larbi A., Hirokawa K., Pawelec G., Bocti C., Lacombe G., Dupuis G., Frost E. H. (2018). Can an Infection Hypothesis Explain the Beta Amyloid Hypothesis of Alzheimer's Disease?. Front. Aging Neurosci..

[cit228] Panza F., Lozupone M., Solfrizzi V., Watling M., Imbimbo B. P. (2019). Time to test antibacterial therapy in Alzheimer's disease. Brain.

[cit229] Soscia S. J., Kirby J. E., Washicosky K. J., Tucker S. M., Ingelsson M., Hyman B., Burton M. A., Goldstein L. E., Duong S., Tanzi R. E., Moir R. D. (2010). The Alzheimer's Disease-Associated Amyloid β-Protein Is an Antimicrobial Peptide. PLoS One.

[cit230] Bourgade K., Le Page A., Bocti C., Witkowski J. M., Dupuis G., Frost E. H., Fülöp T. (2016). Protective Effect of Amyloid-β Peptides Against Herpes Simplex Virus-1 Infection in a Neuronal Cell Culture Model. J. Alzheimer's Dis..

[cit231] Kumar D. K. V., Choi S. H., Washicosky K. J., Eimer W. A., Tucker S., Ghofrani J., Lefkowitz A., McColl G., Goldstein L. E., Tanzi R. E., Moir R. D. (2016). Amyloid-β peptide protects against microbial infection in mouse and worm models of Alzheimer’s disease. Sci. Transl. Med..

[cit232] Kesika P., Suganthy N., Sivamaruthi B. S., Chaiyasut C. (2021). Role of gut-brain axis, gut microbial composition, and probiotic intervention in Alzheimer's disease. Life Sci..

[cit233] Liu S., Gao J., Liu K., Zhang H.-L. (2021). Microbiota-gut-brain axis and Alzheimer's disease: Implications of the blood–brain barrier as an intervention target. Mech. Ageing Dev..

[cit234] Mehta R. I., Mehta R. I. (2023). The Vascular-Immune Hypothesis of Alzheimer's Disease. Biomedicines.

[cit235] Reive B. S., Lau V., Sánchez-Lafuente C. L., Henri-Bhargava A., Kalynchuk L. E., Tremblay M.-È., Caruncho H. J. (2024). The Inflammation-Induced Dysregulation of Reelin Homeostasis Hypothesis of Alzheimer's Disease. J. Alzheimer's Dis..

[cit236] Tang B. L. (2020). Neuropathological Mechanisms Associated with Pesticides in Alzheimer's Disease. Toxics.

[cit237] Fišar Z. (2022). Linking the Amyloid, Tau, and Mitochondrial Hypotheses of Alzheimer's Disease and Identifying Promising Drug Targets. Biomolecules.

[cit238] Jurcău M. C., Andronie-Cioara F. L., Jurcău A., Marcu F., Tit D. M., Paşcalău N., Nistor-Cseppentö D. C. (2022). The Link between Oxidative Stress, Mitochondrial Dysfunction and Neuroinflammation in the Pathophysiology of Alzheimer's Disease: Therapeutic Implications and Future Perspectives. Antioxidants.

[cit239] Kazemeini S., Nadeem-Tariq A., Shih R., Rafanan J., Ghani N., Vida T. A. (2024). From Plaques to Pathways in Alzheimer's Disease: The Mitochondrial-Neurovascular-Metabolic Hypothesis. Int. J. Mol. Sci..

[cit240] Jurisch-Yaksi N., Sannerud R., Annaert W. (2013). A fast growing spectrum of biological functions of γ-secretase in development and disease. Biochim. Biophys. Acta, Biomembr..

[cit241] Müller T., Meyer H. E., Egensperger R., Marcus K. (2008). The amyloid precursor protein intracellular domain (AICD) as modulator of gene expression, apoptosis, and cytoskeletal dynamics—Relevance for Alzheimer's disease. Prog. Neurobiol..

[cit242] Turner P. R., O’Connor K., Tate W. P., Abraham W. C. (2003). Roles of amyloid precursor protein and its fragments in regulating neural activity, plasticity and memory. Prog. Neurobiol..

[cit243] Takei N., Sobu Y., Kimura A., Urano S., Piao Y., Araki Y., Taru H., Yamamoto T., Hata S., Nakaya T., Suzuki T. (2015). Cytoplasmic Fragment of Alcadein α Generated by Regulated Intramembrane Proteolysis Enhances Amyloid β-Protein Precursor (APP) Transport into the Late Secretory Pathway and Facilitates APP Cleavage. J. Biol. Chem..

[cit244] Liu C., Nikain C., Li Y.-M. (2023). γ-Secretase fanning the fire of innate immunity. Biochem. Soc. Trans..

[cit245] Sparling D. P., McCullough N., Pajvani U., Humphrey M. B. (2020). Inhibition of γ-secretase in adipocytes leads to altered IL-6 secretion and adipose inflammation. Adipocyte.

[cit246] Restituito S., Khatri L., Ninan I., Mathews P. M., Liu X., Weinberg R. J., Ziff E. B. (2011). Synaptic Autoregulation by Metalloproteases and γ-Secretase. J. Neurosci..

[cit247] Lee S. H., Sharma M., Südhof T. C., Shen J. (2014). Synaptic function of nicastrin in hippocampal neurons. Proc. Natl. Acad. Sci. U. S. A..

[cit248] Dejaegere T., Serneels L., Schäfer M. K., Van Biervliet J., Horré K., Depboylu C., Alvarez-Fischer D., Herreman A., Willem M., Haass C., Höglinger G. U., D’Hooge R., De Strooper B. (2008). Deficiency of Aph1B/C-γ-secretase disturbs Nrg1 cleavage and sensorimotor gating that can be reversed with antipsychotic treatment. Proc. Natl. Acad. Sci. U. S. A..

[cit249] Pelletier L., Guillaumot P., Frêche B., Luquain C., Christiansen D., Brugière S., Garin J., Manié S. N. (2006). γ-Secretase-Dependent Proteolysis of CD44 Promotes Neoplastic Transformation of Rat Fibroblastic Cells. Cancer Res..

[cit250] Na H.-W., Shin W.-S., Ludwig A., Lee S.-T. (2012). The Cytosolic Domain of Protein-tyrosine Kinase 7 (PTK7), Generated from Sequential Cleavage by a Disintegrin and Metalloprotease 17 (ADAM17) and γ-Secretase, Enhances Cell Proliferation and Migration in Colon Cancer Cells. J. Biol. Chem..

[cit251] Boulton M. E., Cai J., Grant M. B. (2008). γ-Secretase: A multifaceted regulator of angiogenesis. J. Cell. Mol. Med..

[cit252] Sen S., Hallee L., Lam C. K. (2021). The Potential of Gamma Secretase as a Therapeutic Target for Cardiac Diseases. J. Pers. Med..

[cit253] Bi P., Kuang S. (2015). Notch signaling as a novel regulator of metabolism. Trends Endocrinol. Metab..

[cit254] Kim K., Goldberg I. J., Graham M. J., Sundaram M., Bertaggia E., Lee S. X., Qiang L., Haeusler R. A., Metzger D., Chambon P., Yao Z., Ginsberg H. N., Pajvani U. B. (2018). γ-Secretase Inhibition Lowers Plasma Triglyceride-Rich Lipoproteins by Stabilizing the LDL Receptor. Cell Metab..

[cit255] Engin F., Yao Z., Yang T., Zhou G., Bertin T., Jiang M. M., Chen Y., Wang L., Zheng H., Sutton R. E., Boyce B. F., Lee B. (2008). Dimorphic effects of Notch signaling in bone homeostasis. Nat. Med..

[cit256] Zanotti S., Canalis E. (2016). Notch Signaling and the Skeleton. Endocr. Rev..

[cit257] Salhotra A., Shah H. N., Levi B., Longaker M. T. (2020). Mechanisms of bone development and repair. Nat. Rev. Mol. Cell Biol..

[cit258] Pink A. E., Simpson M. A., Desai N., Trembath R. C., Barker J. N. W. (2013). γ-Secretase Mutations in Hidradenitis Suppurativa: New Insights into Disease Pathogenesis. J. Invest. Dermatol..

[cit259] Vellaichamy G., Dimitrion P., Zhou L., Ozog D., Lim H. W., Liao W., Hamzavi I. H., Mi Q.-S. (2021). Insights from γ-Secretase: Functional Genetics of Hidradenitis Suppurativa. J. Invest. Dermatol..

[cit260] Wang Z., Yan Y., Wang B. (2021). γ-Secretase Genetics of Hidradenitis Suppurativa: A Systematic Literature Review. Dermatology.

[cit261] Lee S. M., Han D., Kwon M., Noh H., Lee J. H., Yoon Y., Cho J. Y., Ahn J.-H., Yoon K. (2020). Gamma secretase inhibition impairs HCMV replication by reduction of immediate early gene expression at the transcriptional level. Antiviral Res..

[cit262] Inoue T., Zhang P., Zhang W., Goodner-Bingham K., Dupzyk A., DiMaio D., Tsai B. (2018). γ-Secretase promotes membrane insertion of the human papillomavirus L2 capsid protein during virus infection. J. Cell Biol..

[cit263] Zhang X., Li Y., Xu H., Zhang Y. (2014). The γ-secretase complex: From structure to function. Front. Cell. Neurosci..

[cit264] Otto G. P., Sharma D., Williams R. S. B. (2016). Non-Catalytic Roles of Presenilin Throughout Evolution. J. Alzheimer's Dis..

[cit265] Duggan S. P., McCarthy J. V. (2016). Beyond γ-secretase activity: The multifunctional nature of presenilins in cell signalling pathways. Cell. Signalling.

[cit266] Bergmans B. A., De Strooper B. (2010). γ-secretases: From cell biology to therapeutic strategies. Lancet Neurol..

[cit267] Dunys J., Kawarai T., Sevalle J., Dolcini V., St. George-Hyslop P., Da Costa C. A., Checler F. (2007). P53-dependent Aph-1 and Pen-2 Anti-apoptotic Phenotype Requires the Integrity of the γ-Secretase Complex but Is Independent of Its Activity. J. Biol. Chem..

[cit268] Pardossi-Piquard R., Dunys J., Giaime E., St. Guillot-Sestier M., George-Hyslop P., Checler F., Alves Da Costa C. (2009). p53-Dependent control of cell death by nicastrin: Lack of requirement for presenilin-dependent γ-secretase complex. J. Neurochem..

[cit269] Dovey H. F., John V., Anderson J. P., Chen L. Z., De Saint Andrieu P., Fang L. Y., Freedman S. B., Folmer B., Goldbach E., Holsztynska E. J., Hu K.
L., Johnson-Wood K. L., Kennedy S. L., Kholodenko D., Knops J. E., Latimer L. H., Lee M., Liao Z., Lieberburg I. M. (2001). *et al.*, Functional gamma-secretase inhibitors reduce beta-amyloid peptide levels in brain. J. Neurochem..

[cit270] Anderson J. J., Holtz G., Baskin P. P., Turner M., Rowe B., Wang B., Kounnas M. Z., Lamb B. T., Barten D., Felsenstein K., McDonald I., Srinivasan K., Munoz B., Wagner S. L. (2005). Reductions in β-amyloid concentrations *in vivo* by the γ-secretase inhibitors BMS-289948 and BMS-299897. Biochem. Pharmacol..

[cit271] Wong G. T., Manfra D., Poulet F. M., Zhang Q., Josien H., Bara T., Engstrom L., Pinzon-Ortiz M., Fine J. S., Lee H.-J. J., Zhang L., Higgins G. A., Parker E. M. (2004). Chronic Treatment with the γ-Secretase Inhibitor LY-411,575 Inhibits β-Amyloid Peptide Production and Alters Lymphopoiesis and Intestinal Cell Differentiation. J. Biol. Chem..

[cit272] Siemers E., Skinner M., Dean R. A., Gonzales C., Satterwhite J., Farlow M., Ness D., May P. C. (2005). Safety, Tolerability, and Changes in Amyloid β Concentrations After Administration of a γ-Secretase Inhibitor in Volunteers. Clin. Neuropharmacol..

[cit273] Doody R. S., Raman R., Farlow M., Iwatsubo T., Vellas B., Joffe S., Kieburtz K., He F., Sun X., Thomas R. G., Aisen P. S., Siemers E., Sethuraman G., Mohs R. (2013). A Phase 3 Trial of Semagacestat for Treatment of Alzheimer's Disease. N. Engl. J. Med..

[cit274] Coric V., Van Dyck C. H., Salloway S., Andreasen N., Brody M., Richter R. W., Soininen H., Thein S., Shiovitz T., Pilcher G., Colby S., Rollin L., Dockens R., Pachai C., Portelius E., Andreasson U., Blennow K., Soares H., Albright C. (2012). *et al.*, Safety and Tolerability of the γ-Secretase Inhibitor Avagacestat in a Phase 2 Study of Mild to Moderate Alzheimer Disease. Arch. Neurol..

[cit275] Lewis H. D., Pérez Revuelta B. I., Nadin A., Neduvelil J. G., Harrison T., Pollack S. J., Shearman M. S. (2003). Catalytic Site-Directed γ-Secretase Complex Inhibitors Do Not Discriminate Pharmacologically between Notch S3 and β-APP Cleavages. Biochemistry.

[cit276] Nie P., Vartak A., Li Y.-M. (2020). γ-Secretase inhibitors and modulators: Mechanistic insights into the function and regulation of γ-Secretase. Semin. Cell Dev. Biol..

[cit277] Pajvani U. B., Shawber C. J., Samuel V. T., Birkenfeld A. L., Shulman G. I., Kitajewski J., Accili D. (2011). Inhibition of Notch signaling ameliorates insulin resistance in a FoxO1-dependent manner. Nat. Med..

[cit278] Richter L. R., Wan Q., Wen D., Zhang Y., Yu J., Kang J. K., Zhu C., McKinnon E. L., Gu Z., Qiang L., Pajvani U. B. (2020). Targeted Delivery of Notch Inhibitor Attenuates Obesity-Induced Glucose Intolerance and Liver Fibrosis. ACS Nano.

[cit279] Wang C., Shen J., Yukata K., Inzana J. A., O’Keefe R. J., Awad H. A., Hilton M. J. (2015). Transient gamma-secretase inhibition accelerates and enhances fracture repair likely *via* Notch signaling modulation. Bone.

[cit280] Song C., Zhang J., Xu C., Gao M., Li N., Geng Q. (2023). The critical role of γ-secretase and its inhibitors in cancer and cancer therapeutics. Int. J. Biol. Sci..

[cit281] McCaw T. R., Inga E., Chen H., Jaskula-Sztul R., Dudeja V., Bibb J. A., Ren B., Rose J. B. (2021). Gamma Secretase Inhibitors in Cancer: A Current Perspective on Clinical Performance. Oncologist.

[cit282] Gounder M., Ratan R., Alcindor T., Schöffski P., Van Der Graaf W. T., Wilky B. A., Riedel R. F., Lim A., Smith L. M., Moody S., Attia S., Chawla S., D’Amato G., Federman N., Merriam P., Van Tine B. A., Vincenzi B., Benson C., Bui N. Q. (2023). *et al.*, Nirogacestat, a γ-Secretase Inhibitor for Desmoid Tumors. N. Engl. J. Med..

[cit283] Pont M. J., Hill T., Cole G. O., Abbott J. J., Kelliher J., Salter A. I., Hudecek M., Comstock M. L., Rajan A., Patel B. K. R., Voutsinas J. M., Wu Q., Liu L., Cowan A. J., Wood B. L., Green D. J., Riddell S. R. (2019). γ-Secretase inhibition increases efficacy of BCMA-specific chimeric antigen receptor T cells in multiple myeloma. Blood.

[cit284] Wolfe M. S. (2021). Probing Mechanisms and Therapeutic Potential of γ-Secretase in Alzheimer's Disease. Molecules.

[cit285] Bursavich M. G., Harrison B. A., Blain J.-F. (2016). Gamma Secretase Modulators: New Alzheimer's Drugs on the Horizon?. J. Med. Chem..

[cit286] Weggen S., Eriksen J. L., Das P., Sagi S. A., Wang R., Pietrzik C. U., Findlay K. A., Smith T. E., Murphy M. P., Bulter T., Kang D. E., Marquez-Sterling N., Golde T. E., Koo E. H. (2001). A subset of NSAIDs lower amyloidogenic Aβ42 independently of cyclooxygenase activity. Nature.

[cit287] Weggen S., Eriksen J. L., Sagi S. A., Pietrzik C. U., Ozols V., Fauq A., Golde T. E., Koo E. H. (2003). Evidence That Nonsteroidal Anti-inflammatory Drugs Decrease Amyloid β42 Production by Direct Modulation of γ-Secretase Activity. J. Biol. Chem..

[cit288] Beher D., Clarke E. E., Wrigley J. D. J., Martin A. C. L., Nadin A., Churcher I., Shearman M. S. (2004). Selected Non-steroidal Anti-inflammatory Drugs and Their Derivatives Target γ-Secretase at a Novel Site. J. Biol. Chem..

[cit289] Weber T. A., Lundkvist J., Wanngren J., Kvartsberg H., Jin S., Larssen P., Wu D., Oliveira D. V., Minta K., Brinkmalm G., Zetterberg H., Blennow K., Nordvall G., Winblad B., Portelius E., Karlström H. (2022). γ-Secretase modulators show selectivity for γ-secretase–mediated amyloid precursor protein intramembrane processing. J. Cell. Mol. Med..

[cit290] Crump C. J., Johnson D. S., Li Y.-M. (2013). Development and Mechanism of γ-Secretase Modulators for Alzheimer's Disease. Biochemistry.

[cit291] Ratni H., Alker A., Bartels B., Bissantz C., Chen W., Gerlach I., Limberg A., Lu M., Neidhart W., Pichereau S., Reutlinger M., Rodriguez-Sarmiento R.-M., Jakob-Roetne R., Schmitt G., Zhang E., Baumann K. (2020). Discovery of RO7185876, a Highly Potent γ-Secretase Modulator (GSM) as a Potential Treatment for Alzheimer's Disease. ACS Med. Chem. Lett..

[cit292] Rynearson K. D., Ponnusamy M., Prikhodko O., Xie Y., Zhang C., Nguyen P., Hug B., Sawa M., Becker A., Spencer B., Florio J., Mante M., Salehi B., Arias C., Galasko D., Head B. P., Johnson G., Lin J. H., Duddy S. K. (2021). *et al.*, Preclinical validation of a potent γ-secretase modulator for Alzheimer's disease prevention. J. Exp. Med..

[cit293] Trambauer J., Sarmiento R. M. R., Garringer H. J., Salbaum K., Pedro L. D., Crusius D., Vidal R., Ghetti B., Paquet D., Baumann K., Lindemann L., Steiner H. (2025). γ-Secretase modulator resistance of an aggressive Alzheimer-causing presenilin mutant can be overcome in the heterozygous patient state by a set of advanced compounds. Alzheimer's Res. Ther..

[cit294] Wolfe M. S. (2024). γ-Secretase: Once and future drug target for Alzheimer's disease. Expert Opin. Drug Discovery.

